# Ultraviolet radiation-induced differential microRNA expression in the skin of hairless SKH1 mice, a widely used mouse model for dermatology research

**DOI:** 10.18632/oncotarget.12913

**Published:** 2016-10-26

**Authors:** Ashok Singh, Estelle Willems, Anupama Singh, Irene M. Ong, Ajit K. Verma

**Affiliations:** ^1^ Department of Human Oncology, Wisconsin Institutes for Medical Research, Paul P. Carbone Comprehensive Cancer Center, School of Medicine and Public Health, Madison, WI, 53705, USA; ^2^ Biostatistics and Medical Informatics, Medical Science Center, University of Wisconsin, Madison, WI, 53705, USA

**Keywords:** microRNA, SKH1 hairless mice, SCC, UVR

## Abstract

Cutaneous squamous cell carcinoma (cSCC) is the most common type of non-melanoma skin cancer that can metastasize. The major etiological factor associated with cSCC is Ultraviolet radiation (UVR) with a limited understanding of its molecular mechanism. It was hypothesized that there is a direct effect of UVR on modulation of microRNAs (miRNAs), a novel class of short noncoding RNAs which affects translation and stability of mRNAs. To test the hypothesis, the dorsal skin of the SKH1 mice (6-7 week old) was exposed to acute and chronic doses of UVR. In miRNA array profiling, we found differential expression (log fold change>1) of miR-25-5p between untreated and acute UVR treated (4kJ/m^2^) SKH1 mice skin. However, differential expression (>1 log fold) of miR-144-3p, miR-33-5p, miR-32-5p, miR-1983, miR-136-5p, miR-142-3p, miR-376a-3p, miR-142-5p, miR-3968, and miR-29b-3p was observed between untreated and chronically UVR treated mice skin. Differentially expressed selected miRNAs (miR-32-5p, miR-33-5p, miR-144-3p, and miR-376a-3p) were further validated in real time PCR using miRNA specific primers. Web based data mining, for the prediction of potential miRNA associated gene pathways in miRBase database revealed a link with important pathways (PI3K-Akt, MAPK, Wnt, transcriptional misregulation, and other oncogenic pathway) associated with cSCC. Furthermore, findings of PI3K-Akt pathway genes affected due to chronic UVR were confirmed using cDNA array.

## INTRODUCTION

MicroRNAs (miRNAs) are small endogenous non-protein coding 19-22 nucleotide RNAs. MiRNAs play a vital role in post-transcriptional regulation of various genes relevant in normal and pathological context including various cancers [1, 2]. MiRNAs constitute a novel class of regulators that play an important role during the gene expression. These miRNAs act by direct annealing with a partially complimentary target site within the 3’ untranslated region (3’-UTR) of their target mRNA i.e. seed sequence [3]. Notably, a single miRNA is able to repress up to hundreds of gene transcripts, and in turn be targeted by multiple miRNAs [4]. It has been found that >60% of human protein coding genes are under selective pressure to maintain pairing to miRNAs [5]. Various miRNAs and their signaling components are found to be linked with skin cancer [6, 7]. Identification of novel miRNAs involved in Ultraviolet radiation (UVR)-induced skin cancer may be useful for early diagnostic markers and to check the progression of cancer stages. However, the literature for miRNAs modulated in cutaneous skin cancer by UVR is sparse. Differential miRNA expression has been reported in cutaneous Squamous Cell Carcinoma (SCC) compared to the healthy individuals [8, 9]. Also, it has been reported that there is a specific miRNA expression response due to UVA and UVB radiation in human primary keratinocytes [10]. However, there are limited *in vitro* and *in vivo* studies in connection with UVR and miRNA expression in skin [11–13]. Although, the involvement of miRNAs in the various skin anomalies such as scleroderma, dermatomyositis, psoriasis, epidermal necrolysis, and cancer has been discussed [14].

UVR is a complete carcinogen, and its cumulative exposure in humans leads to SCC. Cutaneous SCC is derived from epidermal keratinocytes, which is the second most common human cancer posing threat to public health with around 250,000 new cases a year [15]. In organ transplant patients, SCC incidences are increased by 60-100% and add additional complexity for disease management and cure [16, 17]. The process of UVR-induced carcinogenesis is directly linked to DNA damage in cells [18, 19]. Lack of information regarding the miRNA expression in response to UVR in the SKH1 hairless mice, a widely used skin carcinogenesis model, prompted us to test the hypothesis that UVR treatment leads to aberrant miRNA expression. In this study, we present for the first time: a) UVR-induced expression profile of miRNAs due to presence (acute and chronic) and absence of UVR in SKH1 mice skin, b) validation of the differentially expressed miRNAs using real time PCR, c) expression pattern of selected miRNAs in UVR-induced SCC samples developed in SKH1 mice, d) bio-informatics analysis of miRNAs and their target pathway, and e) validation of PI3K-Akt pathway involvement due to chronic UVR in the skin of SKH1 mice.

## RESULTS

### UVR induced differential miRNA expression in the skin of SKH1 hairless mice

To determine UVR-induced miRNA modulation SKH1 hairless mice were exposed to either acute (single dose, 4KJ/m^2^) or chronic (4 times X 2KJ/m^2^) UVR. The mice skins were excised for RNA isolation four hours post final UVR exposure. Global miRNA profiling revealed down-regulation of miR-25-5p in acutely UVR treated SKH1 mice skin compared to their untreated littermates. Ten miRNAs (miR-144-3p, miR-33-5p, miR-32-5p, miR-1983, miR-136-5p, miR-142-3p, miR-376a-3p, miR-142-5p, miR-3968, and miR-29b-3p) were differentially expressed (log fold change>1) and down-regulated in chronically treated SKH1 mice compared to untreated controls (Table 1-3).

**Table 1:** Table 1 is showing the differential expression pattern of miRNAs in response to acute UVR (4.0 KJm^2^) compared to no UVR group in the skin of SKH1 mice

miRNAs (mmu)	Chromosomal location	No UVR	Acute UVR	Log FC	p-values	Gene targets
miR-25-5p	chr7: 100093560-100093643 [-]	-0.183	-1.785	-1.602	0.061	19

**Table 2:** Table 2 is showing the differential expression pattern of miRNAs in response to chronic UVR (4 X 2.0 KJm^2^) compared to no UVR group in the skin of SKH1 mice

miRNAs	Chromosomal location	No UVR	Chronic UVR	Log FC	p-values	Gene targets
miR-144-3p	chr11: 78073005-78073070 [+]	0.454	-1.380	-1.833	0.0001	608
miR-33-5p	chr15: 82198122-82198190 [+]	0.645	-1.083	-1.728	0.0003	215
miR-32-5p	chr4: 56895229-56895298 [-]	0.481	-0.788	-1.270	0.0001	323
miR-1983	chr13: 21896918-21897049 [-]	0.186	-1.057	-1.243	0.001	166
miR-136-5p	chr12: 109595327-109595388 [+]	0.575	-0.522	-1.096	0.01	275
miR-142-3p	chr11: 87756864-87756927 [+]	0.471	-0.622	-1.093	0.0001	155
miR-376a-3p	chr12:109723781-109723848 [+]	0.198	-0.889	-1.088	0.008	24
miR-142-5p or miR-142a	chr11: 87756864-87756927 [+]	0.366	-0.692	-1.058	0.0001	498
miR-3968	chr11:115447961-115448060 [-]	-0.010	-1.032	-1.022	0.001	85
miR-29b-3p	chr1: 195037040-195037120 [+]	0.139	-0.870	-1.010	0.0004	326

**Table 3:** Differential expression pattern of miRNAs in acute vs chronic UVR-treated SKH1 mice skin

miRNAs	Chromosomal location	Acute UVR	Chronic UVR	Log FC	p-values	Gene targets
miR-144-3p	chr11: 78073005-78073070 [+]	0.450	-1.380	-1.829	0.0005	608
miR-25-5p	chr7: 100093560-100093643 [-]	-1.785	-0.201	1.583	0.095	19
miR-33-5p	chr15: 82198122-82198190 [+]	0.280	-1.083	-1.364	0.002	215
miR-1983	chr13: 21896918-21897049 [-]	0.139	-1.057	-1.197	0.001	166
miR-3968	chr11: 115447961-115448060 [-]	0.069	-1.032	-1.101	0.001	85
miR-32-5p	chr4: 56895229-56895298 [-]	0.299	-0.788	-1.088	0.001	323

**Figure 1:**
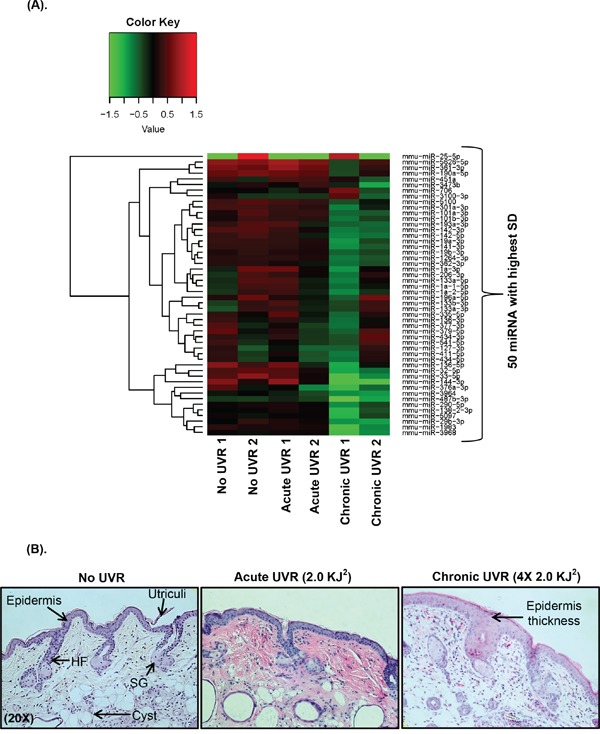
Heat map plot and unsupervised hierarchical clustering of UVR-induced miRNA profiling data **A.** The clustering was performed on all samples, and on the top fifty differentially expressed miRNAs with highest standard deviation (p≤ 0.05). The normalized log ratio values have been used for the analysis. The heat map diagram shows the result of a two-way hierarchical clustering of miRNAs and study samples. The clustering is done using the complete-linkage method together with the Euclidean distance measure. Each row and column represents miRNA and a study sample respectively. The miRNA clustering tree is shown on the left. The color scale red and green illustrates the relative high and low expression level of miRNAs to reference channel respectively. In Figure 1A, a total of two mice (n=2 each group) were used in untreated, acute UVR treated (4.0 KJ^2^), and chronically (4X2.0 KJ^2^) UVR treated SKH1 mice. **B.** Figure B is showing the skin histology following acute and chronic UVR-exposure in hairless SKH1 mice. Mice were exposed single (acute) and four times to UVR (chronic), and were sacrificed at 4hr post last UVR. For histochemistry mice skin specimens (n=3) were processed for HE staining. All HE pictures were captured using Nuance bright field microscope at 20X magnification (abbreviation: HF = hair follicle, SG = sebaceous gland, SD= standard deviation).

Also, six differentially expressed down-regulated miRNAs (miR-144-3p, miR-25-5p, miR-33-5p, miR-1983, miR-3968, and miR-32-5p) were found in chronically UVR treated SKH1 mice compared to acutely treated group. However, miR-25-5p was the only up-regulated miRNAs in chronically treated SKH1 mice compared to acute UVR treatment (Table 1). Histological observation revealed significant epidermal hyperplasia in chronically treated mice skin samples, but not in acutely treated skin (Figure 1A and 1B). Skin hyperplasia indicated the UVR-induced damage in the epidermis of SKH1 mice.

### Validation of miRNAs in qRT-PCR confirms the findings of global miRNA profiling

**Figure 2:**
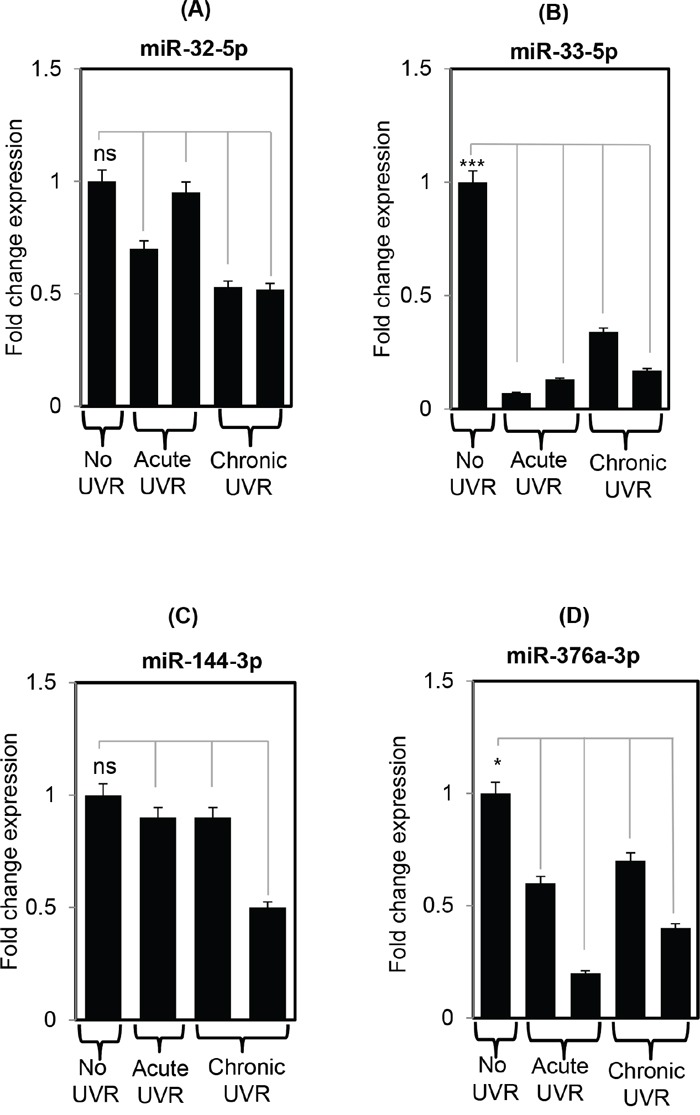
Validation of differentially expressed miRNAs using real time PCR in SKH1 untreated and UVR treated littermates Figure 2 **A-D.** are showing the real time expression pattern of selected miRNAs miR-32-5p, miR-33-5p, miR-144-3p, and miR-376a-3p in untreated, acutely treated, and chronically treated SKH1 mice. The data presented in the each bar diagram is the mean ±SE for each sample in all three groups (n=3 each). The detail of the process is discussed in materials and methods section. The validated miRNA selected for real time PCR based on their average signal intensity value (Hy3 value) >7. Three, two, and one asterisk means highly significant (***), significant (*) or non-significant (ns) respectively.

To confirm the findings of miRNA data, the validation pattern of miR-32-5p, miR-33-5p, miR-144-3p, and miR-376a-3p was analyzed in no UVR, acute, and chronic treated SKH1 skin samples. Expression of miR-33-5p was down-regulated significantly (p<0.05) in acute and chronically UVR-treated mice skin compared to untreated samples. All of these miRNAs (miR-32-5p, miR-33-5p, miR-144-3p, and miR-376a-3p) were down-regulated compared to their untreated littermates, and further confirms the findings of miRNA profiling study (Figure 2).

### Validation of selected miRNAs reveals the down-regulation pattern of miRNAs in UVR-induced cSCC samples from SKH1 mice

**Figure 3:**
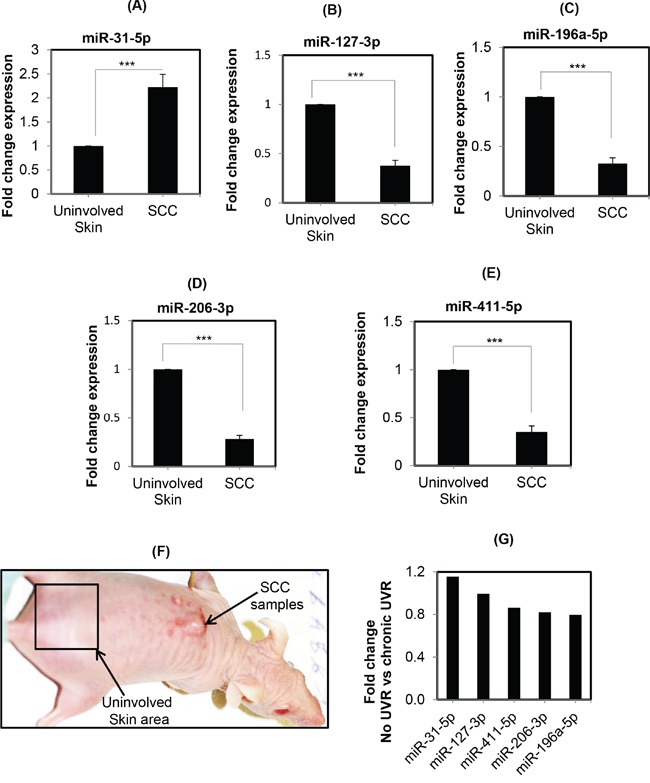
Expression of differentially expressed miRNAs in UVR-induced SCCs from SKH1 mice **A-E.** is showing the expression pattern of miR-31-5p, miR-196-5p, miR-127-3p, miR-206-3p, and miR-411-5p in UVR-induced skin cSCC samples from SKH1 mice. Figures are showing the data from SCC samples collected from UVR-induced carcinogenesis experiments. Each SCC bar diagram is showing the value of triplet repeated from three individual mice (n=3). However, the uninvolved skin (tumor free) samples are from the three mice skin samples (Figure 3F). **F.** is showing one of the representative pictures of UVR-induced cSCC in SKH1 mice. **G.** is expression pattern of these miRNAs comparing chronic UVR with untreated samples in profiling study. Three asterisks mean highly significant (***).

To confirm the consistent pattern and involvement of UVR-induced selected miRNAs in cutaneous SCC samples, the expression of miR-31-5p, miR-196a-5p, miR-206-3p, miR-127-3p, and miR-411-5p were checked in cSCC samples collected from SKH1 mice induced by repeated UVR exposure. Notably, the down-regulation of these selected miRNAs in cSCC samples from FVB mice were reported earlier. The reason for choosing these miRNAs was to further validate the reproducible expression pattern of these miRNAs in SKH1 hairless mice [8, 13]. The cSCC samples were obtained from the dorsal skin of mice after repeated UVR-exposure [20]. It was found that miR-31-5p was significantly (p=0.002) up-regulated (2-fold) in cSCC. However, other miRNAs, miR-196a-5p (p<0.0001); miR-206-3p (p<0.0001); miR-127-3p (p<0.0001); and miR-411-5p (p=0.0002) were significantly down-regulated in three to six UVR-induced cSCC samples from SKH1 mice compared to the uninvolved skin (Figure 3). These results support the consistent reproducible expression pattern of miR-31-5p, miR-196a-5p, miR-206-3p, miR-127-3p, and miR-411-5p in UVR-induced cSCC samples from SKH1 mice.

### Bioinformatics analysis indicates a link of chronic UVR-induced miRNAs to various signaling pathways

**Figure 4:**
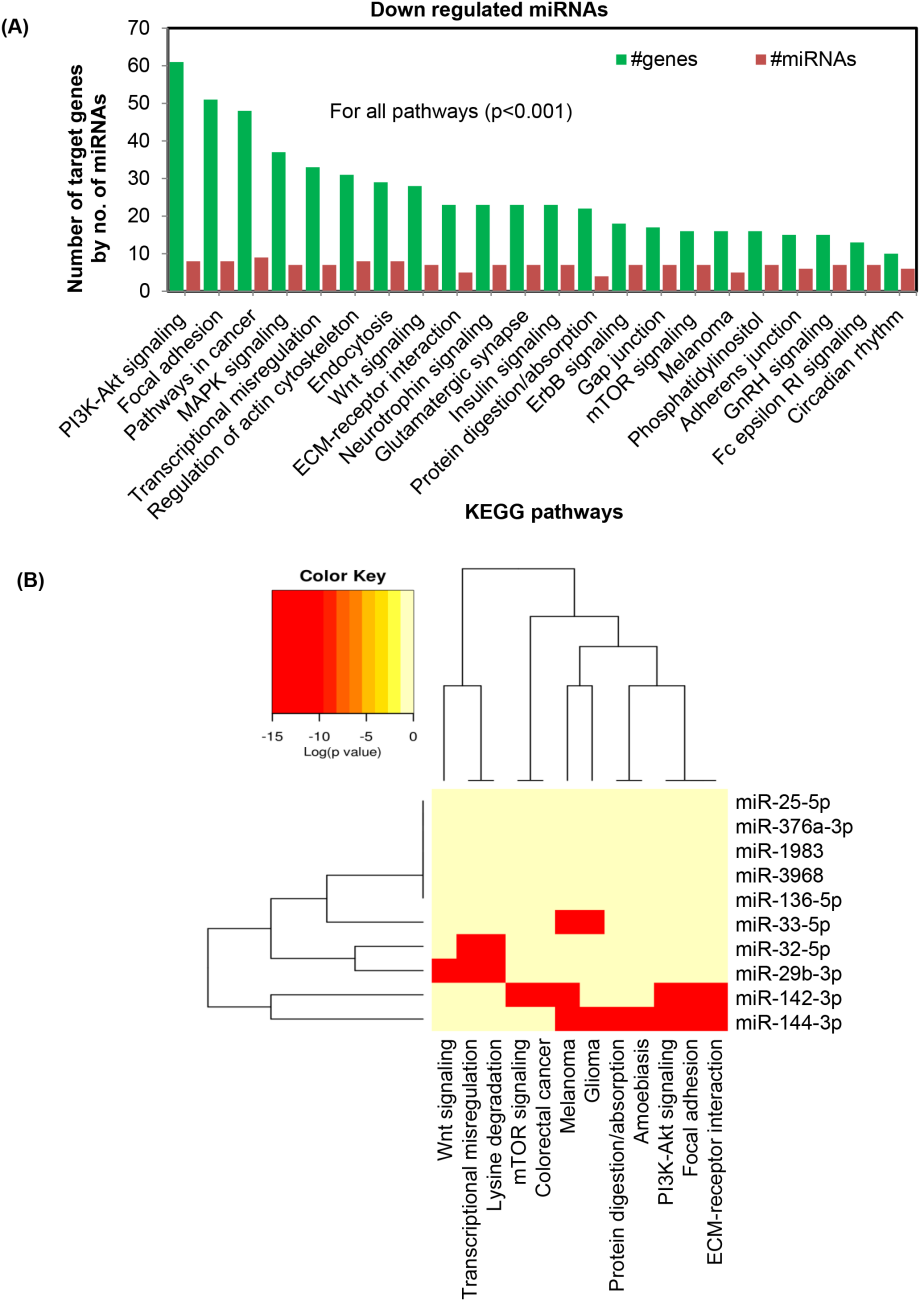
Bioinformatics analysis of differentially expressed miRNAs following chronic UVR exposure in the skin of SKH1 mice Figure 4 **A.** is showing the top KEGG pathways of biological function of the targets of all down-regulated miRNAs between untreated and chronically UVR-treated SKH1 mice. This gene union analysis utilizes the union of targeted genes by the selected miRNAs prior to the statistical calculation. For all these analysis a p-value threshold <0.001 was used. Figure 4 **B.** is showing the targeted pathway cluster of significantly affected top KEGG pathways of biological function of the targets of all down-regulated miRNAs between untreated and chronically UVR-treated skin.

To determine the effect of UVR-induced miRNAs in KEGG molecular pathway, all down-regulated miRNAs affected by chronic UVR in SKH1 mice were used. Combinatorial bioinformatics analysis revealed a set of significantly (p<0.001) affected pathways such as PI3K-Akt, focal adhesion, MAPK, transcriptional misregulation, actin cytoskeleton. Other signaling pathways related to endocytosis, Wnt, ECM-receptor, neurotropin, Glutamatergic, insulin, protein digestion/absorption, ErbB, Gap junction, mTOR, melanoma, phosphatidylinositol, adherens junction, GnRH, Fc epsilon RI, and circadian rhythm are also significantly affected (p<0.001). Notably, PI3K-Akt pathway revealed a total of sixty one targeted genes due to eight significantly (p<0.0001) down-regulated miRNAs in SKH1 mice. Also, targeted pathway clustering analysis revealed that miR-144-3p targets six important signaling pathways, namely, PI3K-Akt, focal adhesion, ECM-receptor interaction, protein digestion and absorption, melanoma, glioma, and amoebiasis. The miR-142-3p can also target ECM-receptor interaction, PI3K-Akt signaling, focal adhesion signaling, and mTOR signaling. The combinatorial effect of ten down-regulated miRNAs (miR-144-3p, miR-33-5p, miR-32-5p, miR-1983, miR-136-5p, miR-142-3p, miR-376a-3p, miR-142-5p, miR-3968, and miR-29b-3p) reveals that a total of 61, 51, 48, and 37 target genes are significantly affected in PI3K-Akt, focal adhesion, cancer pathways, and transcriptional misregulation pathway respectively (p<0.001) (Figure 4).

### PI3K-AKT pathway linked gene analysis

**Figure 5:**
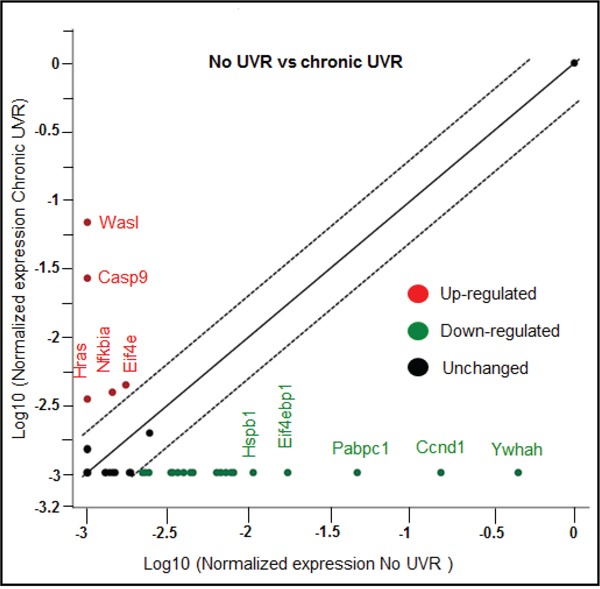
Scatter plot showing up-and down-regulated genes in PI3K-Akt pathway array due to chronic UVR-exposure Figure 5 is showing a scatter plot which compares the normalized expression of every gene on the array between untreated (no UVR) and chronically UVR treated group. Y-axis is showing the top five up-regulated genes (in red). However, the x-axis is showing the five highly down regulated genes (in green). Central line in the scatter plot indicating the most of the unchanged genes in PI3k-Akt pathway. The dotted lines indicate the selected fold regulation threshold i.e., 2 fold. Experiment was repeated two times with same finding.

Our current bioinformatics analysis revealed that there are sixty one genes affected due to a combinatorial effect of miRNAs in PI3K-Akt pathway. To validate the results, a PI3K-Akt focused PCR array was used, which contain eighty four genes along with various other technical controls. In this experiment, the same RNA samples (No UVR and chronically UVR treated samples) for cDNA array, which was used for the miRNA global profiling, were used. The up-regulation of five genes (Eif4e, Nfkbia, Hras, Casp9, and Wasl) and down-regulation of nineteen genes (Ywhah, Ccnd1, Pabpc1, Eif4ebp1, Hspb1, Grb10, Rps6kb1, Itgb1, Mapk1, Gsk3b, Csnk2a1, Hsp90ab1, Mapk8, Grb2, Cdkn1b, Rac1, Rhoa, Gusb, and Rasa1) was observed following chronic UVR (Table 4, Figure 5). Notably, up-regulated genes Hras, Wasl, Ywhah are targeted by miR-1983, miR-32-5p, and miR-33-5p respectively in our present study. Similarly, Mapk8 gene is targeted by three miRNAs miR-32-5p, miR-376a-5p, and miR-29b-3p. All these miRNAs are down-regulated in response to chronic UVR (Table 2-3). Other gene Rac1 is targeted by miR-142a-3p (old name miR-142-3p) in our present study. PI3K-Akt array data further validates our findings of pathway analysis. In summary, the PI3K-Akt pathway is the top pathway affected due to the chronic UVR in the skin of SKH1 mice.

**Table 4:** Table is showing a list of up- and down-regulated PI3K-Akt pathway genes due to chronic UVR-treated in skin of SKH1 mice

Gene description	Gene ID	FC	miRNA in present study
Eukaryotic translation initiation factor 4E	Eif4e	**2.53**	miR-33-3p
Nuclear factor of kappa light polypeptide gene enhancer in B-cells inhibitor, alpha	Nfkbia	**2.71**	—
Harvey rat sarcoma virus oncogene 1	Hras	**3.44**	miR-1983
Caspase 9	Casp9	**26.31**	—
Wiskott-Aldrich syndrome-like	Wasl	**67.25**	miR-32-5p,
Tyrosine 3-monooxygenase/tryptophan 5-monooxygenase activation protein, eta polypeptide	Ywhah	-444.77	miR-33-5p
Cyclin D1	Ccnd1	-148.90	—
Poly(A) binding protein, cytoplasmic 1	Pabpc1	-45.57	miR-33-3p
Eukaryotic translation initiation factor 4E binding protein 1	Eif4ebp1	-17.04	—
Heat shock protein 1	Hspb1	-10.45	—
Growth factor receptor bound protein 10	Grb10	-7.92	—
Ribosomal protein S6 kinase, polypeptide 1	Rps6kb1	-7.66	—
Integrin beta 1 (fibronectin receptor beta)	Itgb1	-7.10	miR-29b-3p, miR-144-3p
Mitogen-activated protein kinase 1	Mapk1	-6.60	—
Glycogen synthase kinase 3 beta	Gsk3b	-6.24	—
Casein kinase 2, alpha 1 polypeptide	Csnk2a1	-4.46	miR-33-3p
Heat shock protein 90 alpha (cytosolic), class B member 1	Hsp90ab1	-4.31	—
Mitogen-activated protein kinase 8	Mapk8	-3.91	miR-32-5p, miR-376a-5p, miR-29b-3p
Growth factor receptor bound protein 2	Grb2	-3.60	—
Cyclin-dependent kinase inhibitor 1B	Cdkn1b	-3.35	—
RAS-related C3 botulinum substrate 1	Rac1	-3.27	miR-142a-3p
Ras homolog gene family, member A	Rhoa	-2.39	—
Glucuronidase, beta	Gusb	-2.26	—
RAS p21 protein activator 1	Rasa1	-2.18	—

In fold change (FC) column the bold and positive values are up-regulated genes due to chronic UVR exposure. However, the negative values are representing down-regulated genes due to UVR exposure. The last column is showing the miRNA in present study targeting the specific gene in PI3K-Akt pathway.

## DISCUSSION

Radiation from sun, particularly its UVR component (280-320 nm) is a complete carcinogen and major etiological factor which leads to skin cancer. Various genes and their signaling components are main target of UVR signaling in skin cancer [21]. However, a little information is known regarding UVR-induced miRNAs and their connection with cSCC. In present study, we have used Kodacel-filtered FS-40 sun lamps as the source of UVR which produces approximately 60% UVB and 40% UVA. This mixture of UVB and UVA mimics the sunlight spectrum reaching to the earth's surface. Also, the 4hr post UVR time points was chosen for miRNA profiling as to detect the early physiological changes in skin micro-environment. Other features such as fast turnover rate and stability due to their specific genomic sequences within the cell or tissue type make them ideal candidates for quick response against altered micro-environment [22, 23].

In this communication, differential expression (log fold change>1) of miR-25-5p was observed between untreated and acute UVR treated SKH1 mice skin. However, a total of ten miRNAs (Table 2) were differentiated expressed due to chronic UVR (4 X 2.0 KJ^2^). Our findings reveal that miRNAs are down-regulated in response to chronic UVR. These may be the initial key players in the process of UVR signaling such as inflammation, reactive oxygen species generation, DNA damage, apoptosis, and proliferation. This is the first report showing the link of UVR and miRNAs in the skin of hairless mice. Two miRNAs miR-136-5p and miR-376a-3p down-regulated in SKH1 mice due to chronic UVR were also suppressed in wild type FVB mice skin due to UVR [13]. This observation indicates a direct link of UVR and modulation of these miRNAs. However, the differences in miRNA profiling data between hairless SKH1 and hairy FVB mice may have a link with the hairless phenotype, less number of adult keratinocyte stem cells, degenerated hair follicle niche, mice genetic background, and abnormal hair cycle. It has been observed that there are ~fivefold less number of CD34^+^/α6-integrin^+^ cells in the epidermal keratinocytes from SKH1 mice compared to FVB [24]. The observation of differential miRNA expression cannot be ignored due to distinct hair morphogenesis, proliferation heterogeneity in epidermal keratinocytes, and hair cycling in hairless mice. It is also clear from our current miRNA profiling and by others that there are differences in miRNA profiling due to UVR intensities, duration of exposure, cell type, and mice background in the study [11–13].

Our profiling study shows that the miR-25-5p was down-regulated due to acute UVR exposure in UVR-sensitive SKH1 mice. A comparison between acute and chronically affected miRNAs indicates that the miR-25-5p was up-regulated (LogFC = 1.58) in chronically treated skin compared to acute UVR. It has been reported that the miR-25 is a direct regulator of p53 gene (encoded by TP53) and negatively regulate the p53 function [25]. We believe that the DNA damage due to UVR in keratinocyte activates TP53 regulated surveillance system. TP53 mediates a response via various miRNAs and genes during the process of carcinogenesis. A direct link between chronic UVR exposure and p53 gene alteration has been discussed in mice skin [26, 27]. Various alterations have been reported in skin due to UVR in a time dependent manner. There was an increase in the number of epidermal cells with wild type p53 protein and at 1hr post UVR exposure and reached maximal levels by 8-12 hrs in SKH1 mice. The alteration pattern of p53 positive nuclei were also followed closely by the p21 (WAF1/CIP1) protein, a responsive gene of wild type p53 [28]. The possibility cannot be ignored that the miR-25 is an initial key regulator of p53 an important tumor suppressor altered during UVR-carcinogenesis.

All the miRNAs with log fold change >1 were down regulated in miRNA profiling study followed by acute and chronic UVR exposure in SKH1 mice compared to their untreated littermates. The suppression of miR-196a-5p, miR-206-3p, miR-127-3p, and miR-411-5p was observed in UVR-induced cSCC samples from SKH1 mice compared to uninvolved SCC free skin. Similarly, the suppression of various mature miRNAs is reported in the present study and many other tumor types [29, 30]. The repression may be due to the direct effect of UVR doses or defects in the biogenesis pathways of miRNA, epigenetic modification, and transcriptional repression [13, 30, 31].

In cSCC samples harvested from SKH1 mice, miRNA-31-5p was up-regulated compared to their uninvolved tumor free skin. The miR-31-5p up-regulation is also reported in clinically well differentiated cSCC samples compared to healthy skin [8]. Our current observations are in corroboration with earlier reports and further strengthens our findings in hairless mice skin [8, 12, 13, 32]. Recently, it has been shown that miR-31 is also implicated in the different stages of hair follicle cycling in the skin via targeting keratin genes in hair follicle [33].

A striking feature of the miRNA is that a single miRNA is capable of targeting hundreds of mRNAs that contain complementary binding sites in their 5’ and 3’UTRs for miRNAs. These target genes may be the part of one or more signaling pathways linked with UVR signaling. We estimated the synergistic role of all down-regulated miRNAs due to acute and chronic UVR exposure to better understand the combinatorial biological function of these miRNAs in skin cancer and cancer associated processes. Using DIANA miRPath v.2.0 tool, we found the pathways related to PI3K-Akt, transcriptional misregulation, focal adhesion, TGF-beta, MAPK, Wnt signaling are targeted by more than one miRNA (4-8 miRNAs) in all of the ten down-regulated miRNAs. In order to further verify the involvement of PI3K-Akt pathway genes in chronic UVR-induced signaling, presence of various up- and down-regulated genes in PI3K-Akt pathway was confirmed. These signaling pathways are linked with skin cancer progression via direct as well as indirect involvement of UVR, miRNAs, and their targeted genes [12, 34–36].

In conclusion, our finding suggests that chronic UVR exposure has pleotropic effects in the epidermal niche, which can modulate various miRNAs and their target genes (See supplementary Tables 1 and 2 for detail miRNA targets and common target in three databases TargetScan, DIANA, and miRDB). Most of these miRNAs are down-regulated with acute and chronic UVR doses in the skin. Synergistically, all the down-regulated miRNAs following chronic UVR modulate various genes implicated in biological pathways linked to skin homeostasis and cancer. Present miRNA profiling observation provides novel insight for future exploration of miRNAs in connection with UVR and their functional implication in skin carcinogenesis. Due to vast regulatory potential, tissue specific, and disease specific expression patterns, the miRNAs can also be used for diagnosis marker and prediction of UVR damage in pre-malignant skin tissues. Our results may be valuable for future design of studies to decipher the precise role of the individual miRNA in the various stages of UVR-induced carcinogenesis.

## MATERIALS AND METHODS

### Mice, UVR source and treatments

Female SKH1 hairless mice were purchased from Charles River Laboratory (Wilmington, MA). SKH1 mice were housed in groups of two to three in plastic bottom cages in light, humidity, and temperature-controlled rooms; food and water were available *ad libitum*. The animals were kept in a normal rhythm of 12h light and 12h dark periods. The UVR source used in this study was Kodacel-filtered FS-40 sun lamps (approximately 60% UVB and 40% UVA). UVR dose was measured using UVX-radiometer (UVP, Upland, CA). Kodacel filters were purchased from Unique Photo Inc. (Fairfield, NJ), and UVR lamps from National Biologicals Corporation (Beachwood, OH). Mice were used for experimentation starting at 5 to 6 weeks of age. SKH1 mice were exposed to acute UVR treatment once (4kJ/m^2^) and 4 hrs post-UVR treatment mice were sacrificed for preparation of epidermal skin RNA samples. There were a total of three groups with three mice in each group: 1) untreated mice, 2) acute UVR-treated mice (4kJ/m^2^), and 3) chronically treated SKH1 mice [4X 2kJ/m^2^ (Monday, Wednesday, Friday and Monday)]. Cutaneous SCC samples induced by UVR were collected from SKH1 mice skin. A detail about the UVR-carcinogenesis is discussed before [20]. All of the animal protocols were approved by the University of Wisconsin Research Animal Resources Committee in accordance with the NIH Guideline for the Care and Use of Laboratory Animals.

### RNA isolation, sample preparation for miRNA profiling

The dorsal epidermal skin of the mice was removed for total RNA isolation. RNA was isolated using the Trizol (Invitrogen, USA) method. The concentration of RNA was determined by measuring the absorbance at 260nm (A260) in a NanoDrop spectrophotometer. The quality of the total purified RNA for miRNA profiling was evaluated using an Agilent 2100 Bio analyzer (Agilent Technologies, Palo Alto, CA, USA), and also confirmed on agarose gel for its integrity. A total of six mice were used in profiling study. In each group (untreated, acute, and chronic treated) RNA samples from two mice skin (n=2 each group) were used for separate global miRNA profiling.

### Profiling of miRNA array

Global miRNA array profiling was conducted at Exiqon Services, Denmark. Briefly, 750 ng total RNA from both sample and reference was labeled with Hy3™ and Hy5™ fluorescent label, respectively, using the miRCURY LNA™ miRNA Hi-Power Labeling Kit, Hy3™/Hy5™ following the procedure described by the manufacturer. There were 1157 spots on the miRNA array slide. The Hy3™-labeled samples and a Hy5™-labeled reference RNA sample were mixed pair-wise and hybridized to the miRCURY LNA™ miRNA Array 7th Gen, which contains capture probes targeting all miRNAs for mouse registered in miRBASE 18.0. The hybridization was performed according to the miRCURY LNA™ miRNA array instruction manual using a Tecan HS4800™ hybridization station (Tecan, Austria). After hybridization the microarray slides were scanned using the Agilent G2565BA microarray scanner system and image was analyzed using the ImaGene® 9 Software. The quantified signals were background corrected [37] and normalized using the global Lowess regression algorithm.

### Primers for miRNA

To validate the global miRNA profiling results, miRNAs were selected based on their up and down regulation compared to their untreated controls. The miRNAs chosen for validation having average Hy3 values (intensity of spot) >7, and fold change values >1. MiRNA mouse primers miR-31-5p, miR-709, miR-322-5p, miR-32-5p, miR-33-5p, miR-376a-3p, miR-144-3p, miR-136-5p, U6 snRNA (internal control) were procured from Exiqon. Primers were reconstituted in 220uL of nuclease free water and mixed properly before use in real time PCR. A detailed list of miRNA primers and their target sequences is available online (http://www.exiqon.com/plate-layout-files).

### cDNA synthesis, real-time PCR

Reverse transcription reaction was set up according to instruction manual using cDNA synthesis kit (Exiqon, miRCURY LNA^TM^ Universal RT miRNA PCR, Polyadenylation and cDNA synthesis kit II, product code 203301). The template RNA 5ng/μL, 5X reaction buffer (2μL), enzyme mix (1μL), and nuclease free water was added together to make final volume total of 10μL. The reaction mixture was gently pipetted, mixed thoroughly and spun down. For cDNA synthesis reaction, PCR settings were as follow: 60 min at 42°C, heat inactivation of reverse transcriptase for 5 min at 95°C, and stored at 4°C.

Before using cDNA for RT-PCR reaction, the required amount of cDNA was diluted 80 times (1:80 dilution) in nuclease free water and used immediately. For running the RT-PCR reaction, a working solution was prepared with ExiLENT SYBR Green (Exiqon, product code 203402) master mix (5 μL), PCR primer mix (1 μL), diluted cDNA template (4 μL) and the total volume was adjusted to 10uL. The reaction was set up in Bio-Rad real time PCR. The RT-PCR programme was set up as follows: 95°C for 10 min, 40 amplification cycles at 95°C, 10 sec at 60°C, 1 min ramp rate 1.6C/sec. All the reactions were set up using nuclease free PCR tubes. However, the cDNA for focused cDNA array (PI3K-Akt pathway) was synthesized using RT2 First Strand kit (Cat# 330401, Qiagen, Germany) as per the manufacturer's protocol. We used a total of 0.5 μg of total RNA from untreated and chronically UVR treated SKH1 skin for real-time expression in PI3K-Akt focused PCR array.

### Analysis of miRNA expression

To analyze the fold change pattern of miRNA, a cycle to threshold (Ct) value was assigned automatically at the beginning of the logarithmic phase of real time PCR. Difference in Ct value of control (endogenous control U6 snRNA) and miRNA were used to determine the relative gene expression or fold changes. This method relies on comparing the differences in Ct values obtained for the target miRNA and control. First, the Ct values for all the samples are extracted and delta Ct is calculated as the difference in Ct between miRNA target and endogenous control [ΔC_T_ = C_T_ (target miRNA) - C_T_ (endogenous control)]. Secondly, the ΔΔC_T_ is calculated [ΔΔC_T_ = ΔC_T_(sample of interest) - ΔC_T_ (control sample)]. Normalization of target miRNA target gene expression in the sample of interest is determined as 2^-ΔΔCT^. Atfinal, the normalized expression level of control sample is set to 1 and change in target miRNA gene expression is determined as: Fold change in target miRNA expression = 1- normalized target miRNA expression in sample of interest.

### Target prediction and pathway analysis

For prediction of miRNA targets, we used freely available bioinformatics tools such as DNA intelligent analysis or DIANA (http://diana.cslab.ece.ntua.gr/), MIRANDA, Target Scan (http://www.targetscan.org/), and miRNA database or miRDB (http://www.mirbase.org/). These bioinformatics tools predict the miRNA and its putative targets on the basis of sequence complementarity in 5’ and 3’ UTRs along with various sequence based mathematical algorithms. We also used DIANA miRPath v.2.0 for investigating the combinatorial effects of UV modulated miRNAs in various biologically relevant pathways [38]. The DIANA-miRPath web-server was able to predict miRNA targets (in coding or 3’-UTR regions) using the DIANA-microT-CDS algorithm. The default settings p-value 0.001 and threshold score 0.8 was used. These observed predicted and/or validated interactions were subsequently combined with sophisticated merging and meta-analysis algorithms [38].

### Gene expression analysis using pathway focused array

Pathway-focused gene expression profiling was done using a 96-well mouse PCR array, RT2 Profiler PCR array (PAMM-058Z, Mouse PI3K-Akt PCR Array, Qiagen, USA). This RT2 Profiler PCR array contains a total of 96 well with eighty-four PI3K-Akt genes and other internal and technical controls (positive, negative, DNA contamination controls). The genes in the array are involved in various processes of PI3K-Akt signaling. A detail list of the PCR array genes is available on SABiosciences website (http://www.sabiosciences.com/rt_pcr_product/HTML/PAMM-058Z.html). RNaseZap® was used to avoid any RNase contamination. Diluted cDNA aliquot, 0.5 μg total RNA, nuclease free molecular grade water, and RT2 SYBR® green master mix (Cat#330504, Qiagen, USA) was mixed as per manufactures protocols and pipetted to the 96-well array plate. The reaction was set up in Bio-Rad real time PCR iCycler (96 CFX, Bio-Rad, USA). The RT-PCR programme was set up as followed: 95°C for 10 min, 40 amplification cycles at 95 °C for 15 s, and 1 min at 60°C.

### Data analysis for PI3K-Akt PCR array

The cycle threshold (Ct) values for each sample were given automatically by the Bio-Rad 96-CFX iCycler according to the amplification curves. The selected threshold was 20.0 and the baseline cycles were 2–10. The PCR array expression data analysis was performed using the ΔΔCt method. The data was analyzed using the online web resources available to the SABiosciences company (Qiagen, USA) web portal http://www.qiagen.com/us/shop/genes-and-pathways/data-analysis-center-overview-page/). Changes in gene expression pattern were presented as a fold change increase or decrease. The data were normalized, across all plates, to the housekeeping gene glyceraldehydes-3-phosphate dehydrogenase (Gapdh).

### Statistical analysis

Student t-test was used to determine the difference between untreated and UVR treated samples for validation (no UVR, acute, and chronic UVR) in SKH1 skin. Differences in expression level of miRNAs in uninvolved skin (skin without tumor) and cSCC were examined using unpaired t-test. p≤ 0.05 was considered to be statistically significant. Limma [39] and differential expression analysis [40] packages were used to determine differentially expressed genes in the following comparisons: no UVR vs. acute, no UVR vs. chronic UVR, and acute vs. chronic UVR. Cut-off criteria in all the expression table (1-3) was p-value < 0.05 and |log_2_ fold-change (FC)| > 1.

## SUPPLEMENTARY MATERIALS TABLES







## Abbreviations

UVR: ultraviolet radiation
SCC: squamous cell carcinoma
microRNA: miRNA
