# Supplementary material for: Ultraviolet radiation-induced differential microRNA expression in the skin of hairless SKH1 mice, a widely used mouse model for dermatology research
Source: Oncotarget. 2016 Oct 26;7(51):84924–37. doi: 10.18632/oncotarget.12913 (PMC5356709; doi:10.18632/oncotarget.12913)
Supplement: Supplementary file 1 [file oncotarget-07-84924-s001.pdf]

Ultraviolet radiation-induced differential microRNA expression in the skin of hairless SKH1 mice, a widely used mouse model for dermatology research

SUPPLEMENTARY TABLES

Supplementary Table S1: The Table is showing the list of common miRNA target genes in three online miRNA data bases (TargetScan, DIANA, and miRDB, left column) for differentially affected miRNAs in SKH1 mice.

See Supplementary file S1

Supplementary Table S2: Online miRDB data bases TargetScan, DIANA, and miRDB (left column) showing the list of predicted target genes (right column) of differentially affected miRNAs in SKH1 mice.

See Supplementary file S2
