# Supplementary material for: Ultraviolet radiation-induced differential microRNA expression in the skin of hairless SKH1 mice, a widely used mouse model for dermatology research
Source: Oncotarget. 2016 Oct 26;7(51):84924–37. doi: 10.18632/oncotarget.12913 (PMC5356709; doi:10.18632/oncotarget.12913)
Supplement: Supplementary file 2 [file oncotarget-07-84924-s002.docx]

| **miRNA (mmu)** | **Target genes in TargetScan, DIANA, and miRDB databases** |
| --- | --- |
| **miR-32-5p** | **Myo1b, Papd7, Ugp2,** 4932438a13rik, Aars, Adam10, Adamtsl1, Adamtsl3, Ankrd44, Appl1, Arid1b, Arrdc3, Atxn3, Avl9, Bcl11a, Bcl11b, Bsdc1, Btg2, C030046e11rik, Ccnjl, Cd69, Cdca7l, Cep41, Cic, Cldn11, Cog3, Col1a2, Cpeb3, Dcaf6, Ddc, Ddx3x, Dkk3, Dmxl1, Dnajb9, Dpp10, Dpy30, Dsc2, Dynlt3, Efr3a, Elovl4, Eomes, Ergic2, Exoc5, Fam135a, Fam160b1, Fam19a1, Fbn1, Fbxw7, Fmr1, Fnip1, Foxn2, G3bp2, Gata2, Gfpt2, Glyr1, Gm10639, Golga4, Gpbp1l1, Gpr180, Gpr98, Gramd3, Grhl1, Gsta2, Hand2, Herpud2, Hipk3, Hivep1, Hnf1b, Hps6, Iqgap2, Isca1, Itgav, Itpr1, Kat2b, Klf4, Klhl14, Klhl29, Lhfpl2, Luzp1, Man2a1, Map1b, Map2k4, Mapk8, Mia3, Morc3, Mycbp2, Myo5a, Nckap5, Necap1, Nlk, Nova1, Nox4, Osbpl8, Pcdh11x, Pcgf3, Pcmtd1, Pcolce2, Peak1, Per2, Phlpp2, Phtf2, Pitpnm2, Pkdcc, Plekha1, Pof1b, Pp2d1, Ppcs, Ppp1r37, Prkar1a, Prkar1b, Ptar1, Ptger4, Ptprj, Ptpro, Rab14, Rad21, Rbpms2, Rev3l, Rgs17, Rgs3, Rhpn2, Rsbn1, Scn8a, Sgpp1, Sim2, Slc12a5, Slc24a3, Slc25a32, Slx4, Snn, Snx13, Socs5, Spryd4, Srpr, Ssfa2, Syn2, Synj1, Tagap, Tbl1xr1, Tef, Tgif1, Tmem229a, Tob1, Tob2, Tulp4, Ube2z, Usp28, Wasl, Wrnip1, Wwp2, Xrn1, Zeb2, Zfc3h1, Zfyve21 |
| **miR-144-3p** | **Cav2, Ccng2, Eif4g2, Nfe2l2, Nr2f2, Pafah1b1, Sco1, Tek, Tspan3, Ube2g1, Zranb2**, 9930021j03rik, Abca1, Abi1, Acbd5, Adamts15, Ahdc1, Aldh1a3, Antxr2, Arid1a, Arid2, Asap2, Atp2b1, Atp2b2, B230219d22rik, Bach2, Brpf1, Cask, Ccdc85a, Ccdc88a, Cct6a, Crebrf, Dlg5, Dok4, Dtwd1, E2f8, Eea1, Ehmt1, Ets1, Fam178a, Fam196a, Fam60a, Fam76b, Fat4, Fbn2, Fbxl3, Fbxw11, Fmr1, Fndc3a, Galnt3, Gdf10, Gm5531, Gspt1, Hdgfrp3, Herpud1, Hif1a, Hnrnpf, Hnrnpu, Impact, Ireb2, Itsn2, Kat6a, Khdrbs3, Kitl, Klf8, Limch1, Magt1, Mapk6, Med14, Med4, Megf9, Mob4, Msx1, Nacc2, Pank1, Pcdh18, Pcsk5, Phtf2, Pik3c2a, Pla2g4a, Plat, Plekhg1, Pnrc1, Ppp1r16b, Prr11, Ptp4a1, Ptpn12, Ptpn9, Pura, Rarb, Rc3h2, Rfx3, Rin2, Rnaseh1, Sall1, Scfd1, Scn1a, Sec24a, Senp7, Sh3tc2, Slc12a2, Slc16a12, Slc23a2, Slc25a36, Slc5a7, Smad4, Smoc1, Son, Sorcs3, Sp4, Ss18, Ssb, St18, Stard8, Strn, Suclg2, Sumo3, Tbl1xr1, Tet2, Tfap4, Thap1, Tjp1, Tmem184c, Tmem65, Tnfsf11, Tnrc6a, Ttn, Uba2, Ube2d3, Ubr3, Ubxn7, Uchl3, Usp42, Vkorc1l1, Vps4b, Wdfy3, Wif1, Zbtb18, Zbtb21, Zdhhc17, Zfx, Zzz3 |
| **miR-142-5p**  **(miR-142a-5p)** | **Btf3l4, Ccng2, Mal2, Rhoc,** A830018l16rik, Adamts1, Aff4, Ai467606, Arap2, Arhgef12, Arid2, Atxn7l2, Bai3, Bicd1, Btg3, C77370, Camsap2, Capn7, Caprin2, Cbln4, Cbx3, Cdk17, Cntn1, Commd2, Diap2, Dio2, Dnajc25, Efcab14, Elavl4, Etv1, Fam126b, Fam199x, Fam19a1, Fbxl3, Gas7, Gdnf, Gopc, Gpr75, Grsf1, Herpud1, Hipk1, Hnrnph3, Hook3, Igf2bp3, Iltifb, Kif13a, Kif5c, Kitl, Klf10, Klf11, Lmx1a, Lrp2, Map3k13, Mbd2, Med14, Med28, Myo1d, Nck2, Necab1, Nedd1, Npat, Pcgf3, Pds5b, Prpf40a, Prpf4b, Ptpn4, Rab6b, Rap1a, Rev3l, Rhoa, Rhoq, Rnf146, Rnf165, Rnh1, Robo1, Rsf1, Setd2, Sgce, Slain1, Slc18a2, Srsf6, Stag1, Stau1, Stc1, Synj1, Tiam1, Tmem245, Tmx3, Uba3, Ube2a, Ube2d1, Vmp1, Vps54, Wwp1, Zc3h12c, Zfp275, Zfp503, Zfpm2 |
| **miR-33-5p** | **Hadhb, Slc25a25, Ywhah,** Abca1, Arid5b, Braf, Cntn4, Cybrd1, Dcun1d5, Glcci1, Hmga2, Mlxip, Naa15, Naa30, Pdgfra, Pim1, Rap2a, Rgs7bp, Rpp14, Scn8a, Sema7a, Setd7, Six4, Slc12a5, Tmem86a, Tph2, Vcan, Zfp281 |
| **miR-1983** | **Srsf2,** Arrdc3, Bach1, Clock, Crkl, Gltpd1, Hipk3, Hnrnpll, Pcsk2, Pi4k2a, Ppm1h, Rab8b, Rnf44, Spty2d1, Stc2, Ube2g1, Zmynd11 |
| **miR-3968** | **Ralgapb,** Cxxc5, Foxp2, Gclc, Hnrnpu, Mtpn, Nbr1, Nfia, Pip5k1a, Rbp4, Rc3h1, Sema4a, Spop, Srsf3, Txndc17, Ubqln1 |
| **miR-142-3p** | Apc, Arntl, Baz1a, Brwd3, Cxadr, Dirc2, Egfl6, Eml4, Foxo4, Fyco1, Hectd1, Inpp5a, Itgav, Kat2b, Kif5b, Marcks, Mbd6, Mlxip, Morf4l2, Myh10, Ptpn23, Rgl2, Rictor, Rlf, Sik1, Strn3, Taok1, Tgfbr1, Trpc3, Twf1, Utrn, Wasl, Zbtb41, Zeb2 |
| **miR-136-5p** | Arf6, Atrn, Braf, Cbx4, Ccng1, Cntn2, Crebzf, Fmr1, Gria1, Ino80, Jazf1, Mllt3, Mtpn, Ppp2r2a, Rasl10b, Rnf139, Rpusd4, Sema4c, Wdr43 |
| **miR-29b-3p** | Adamts10, Adamts2, C77370, Col1a1, Col2a1, Col4a5, Col5a1, Col5a2, Col5a3, Col7a1, Dnmt3a, Fbn1, Ifi30, Igf1, Nav1, Nfia, Nsd1, Sestd1, Tet1, Tet2 |
| **miR-376a-3p** | Atp2a2, Bmp2, Kmt2a, Stc1 |
| **miR-25-5p** | Actr1a |

**Table S1: The Table is showing the list of common miRNA target genes in three online** **miRNA data bases (TargetScan, DIANA, and miRDB, left column) for differentially affected miRNAs in SKH1 mice.** In Table S1, the genes in bold black and underlined are the target genes common in all three databases, however rest of the genes are common in any two databases. For TargetScan, the order of genes is based on number of 3P-seq tags + 5 (high to low value with a cut off at 1000 genes). However, in DIANA and miRDB database the criterion of target gene section is based on miTG score (high to low value with a threshold ≥ 0.8) and target score (high to low value with cut of at 0.8) respectively.
