# Supplementary material for: Ultraviolet radiation-induced differential microRNA expression in the skin of hairless SKH1 mice, a widely used mouse model for dermatology research
Source: Oncotarget. 2016 Oct 26;7(51):84924–37. doi: 10.18632/oncotarget.12913 (PMC5356709; doi:10.18632/oncotarget.12913)
Supplement: Supplementary file 3 [file oncotarget-07-84924-s003.docx]

**Legends: S2 Table:** **Online** **miRDB data bases TargetScan, DIANA, and miRDB (left column) showing the list of predicted target genes (right column) of differentially affected miRNAs in SKH1 mice.** For TargetScan, the order of genes is based on number of 3P-seq tags + 5 (high to low value with a cut off at 1000). However, in DIANA and miRDB database the criterion of target section is based on miTG score in (high to low value with a threshold ≥ 0.8) and target score (high to low value with cut of at 0.8) respectively.

| miRNA databases | Differentially expressed SKH1 miRNAs and their predicted target genes | |
| --- | --- | --- |
| (miR-25-5p) | | |
| TargetScan | Trpv2, Xrcc5, Ssr1, Anxa6, Actn1, Id1, Lpl, Cotl1, Sox12, Tubb6, Actr1b, Larp1, Bcam, Cybrd1, Poldip2, Trnp1, Agfg2, C2orf72, Errfi1, Cltb, Slc35b2, Eif3k, Galnt10, Por, Agtrap, Malsu1, Tm9sf4, Znf282, Tceb3, Ankrd52, Repin1, Galnt2, Tram2, Sh3rf1, Sox4, B3gnt1, Hdgfrp3, Asxl1, Kctd15, Cyb5b, Marveld1, Pagr1, Pagr1, Epha2, Timmdc1, Tgfbr2, Ctdnep1, Npdc1, Sort1, Mmab, Gpx8, Tmem37, Pim1, Hif1a, Wdr83os, Nptx1, Cbx2, Znf24, Slc25a26, Sco1, Ankrd13a, Ube2v1, Tmem189-Ube2v1, Tmem189, Isoc2, Aacs, Hif1an, C1orf198, Foxm1, Bod1, Eif4a3, Grpel1, Tspan9, Mex3b, Sec14l1, Arl3, Vps53, Ablim1, Ctdsp1, Six1, Mgea5, Pigk, Mapk14, Dynll2, Itprip, Mrps27, Ptk2, Wdr13, Ncs1, Znf579, Kiaa0141, Mms19, Styxl1, Junb, Map7d1, Poc1a, Cdh13, B4galt5 | |
| DIANA | BC049702, Wdr62, Rhbdd2, Ppfia4, Ripk4, Actr1a, Npdc1, Gm10463 | |
| miRDB | Actr1a, Sptbn4, Fxr2 | |
| (miR-144-3p) | | |
| TargetScan | | Eif4g2, Sumo3, Ube2d2, Tspan3, Ube2g1, Nr2f2, Ncbp2, Zranb2, Uchl3, C5orf24, Hdgfrp3, Cct6a, Nfe2l2, Sco1, Rasa1, Pafah1b1, Rnf146, Luzp6, Cav2, Ccng2, Tek |
| DIANA | | Sorcs3, Pura, Srsf10, Zfp36l2, Brpf1, Nacc2, Myo1e, Pafah1b1, Rarb, Usp42, Msx1, Ptpn9, Bmpr1b, Tet2, Spred1, Zcchc2, Sall1, Map7d1, Dmd, Fam126b, Mef2a, Tnpo1, Thap1, Atxn1, Rgma, Ccng2, St18, Rc3h2, Arid1a, Ppp3r1, Fmn2, Nsd1, Tet1, Plekhg1, Cux1, Zfx, Ets1, Atp2b1, Fndc3a, Fat4, Bach2, Nfe2l2, Appbp2, Sec24a, Sap30l, Ube2d2a, Ube2d1, Fbn2, Cachd1, Rfx3, Bbc3, Six4, Nova1, Eif4g2, Ttn, Ikzf4, Zbtb18, E2f8, Cxcl12, Frs2, Mapk6, Zeb1, Ssx2ip, Ebf1, Zfp800, Son, Ehmt1, Slc12a2, Scamp1, Med12l, Nr2f2, Spag11b, Med14, Abca1, Pcdh18, Jph1, Ppp1r16b, Fbxo32, Aldh1a3, Herpud1, Sh3tc2, Uba2, Tfap4, Arid2, Zbtb38, CG1273, Pank1, Pla2g4a, 1190002N15Rik, Cdc14a, Nav3, Fam76b, Zfhx4, Pnrc1, Sema6d, Socs7, Scn8a, Col10a1, Rgl1, Abtb2, Magi1, Fmr1, Gm10175, Mob4, Hnrnpf, Ubr3, Rin2, Shank2, Plxnc1, Dgcr2, Hdac9, Eea1, Ssb, Smoc1, Crebrf, Zc3h12c, Atp1b1, Gnb4, Htra3, Dennd1a, Phtf2, Ube2g1, Pik3c2a, Kcnh7, Mycn, Scn1a, Mycl, Ube2d3, Phf3, Spast, Pknox1, Fam60a, Erbb2ip, Zbtb20, Pde4a, Nptx1, Kat6a, Impact, Ubn2, Cdh20, Nr3c1, Pcsk5, Vkorc1l1, Zfp148, Myo9a, Ino80d, Zfhx3, Pptc7, Kcnd2, Ylpm1, Arhgap20, Lhx1, Nlk, Dcbld2, Adamts15, Atxn1l, Megf9, Idh2, Fst, Gdf10, Adamts17, Acbd3, Zfp120, Nyap2, Zbtb21, Clk4, Zfp422, Gm10231, Map3k4, Pfkl, Dtna, Slitrk4, Ccdc88a, Met, Asap2, Slc5a7, Zdhhc21, Strn, Slc7a11, Grm5, Lrrc39, Gm8005, Slc4a10, Adamtsl3, Ipo8, Fgfr1op, Gm21092, Slc25a36, Tfrc, Ccdc85a, Arhgap26, Phf6, Limch1, Sel1l, Rgs17, Shisa6, Gpr85, Fbxl3, Plat, Skor1, Hivep3, Kcnc4, Zfp804a, Pygo1, Ss18, Smad4, Med13, Gm5531, Trappc8, Tbx1, Robo2, Cspp1, Cpeb2, Chek1, Heg1, Mkl2, Rnf111, Tek, Slc23a2, B230219D22Rik, Zeb2, Atrx, Afap1, Cadm2, Tnrc6a, Mbnl2, Usp47, Camta1, Zranb2, Qk, Magi2, Kbtbd2, Celf2, Pthlh, Gm8068, Parn, Tab3, Pak7, Gm21977, Pcdh10, Abhd17b, Wdfy3, Bbx, Fbxw11, Nacc1, Nr6a1, Ap1g1, Fam178a, Elavl2, Ptgfrn, Gm15091, Gm15127, Kdm3a, Gm15093, Plekha7, Cdk8, Zfml, Gm15080, Gm8138, Gm8122, Gm15085, Trove2, Khdrbs3, Hnrnpu, Gm15114, Tnrc6b, Gm10439, Ott, Pappa, Hoxa10, Eif5, Sec22c, Abi2, Scfd1, Itgb8, Tnfsf11, Tspan12, Otud4, Sgpp1, Wif1, Cd160, Tgif2, Hif1a, Cds1, Ireb2, Aspn, Tsc22d2, Gm8180, Tmem184c, Flrt2, Htr2c, Fam196a, Cbx4, Gm15128, Lifr, Marcks, Gm17079, Ppig, Zfp746, Gm6482, Srek1, Hspbap1, Gm15107, Efna5, Smarca1, Rasgrp1, Trio, Ammecr1, Vps4b, Ptpn12, Rab1, Gm15097, Gm8165, Gm6401, Elk3, Kif2a, Gata3, Galnt3, Tenm3, Ube2a, Gm7951, Gm8020, Ppp2r2a, Gm17175, Fam199x, Gm3327, Gm7980, Sgk1, Ankrd17, Fgd6, 9930021J03Rik, Rnaseh1, Sumo3, Bmp2k, Gm7945, Gm16506, Bzw1, Suclg2, Mapk8, Gm17124, Dr1, Gm17026, Gm7970, Dtwd1, Cdc73, Snn, Abhd17c, Mitf, Ufm1, Mtmr1, Mbnl1, Med4, Nfib, Clint1, Gja1, Tspan3, Tshz3, Cxxc4, Btbd3, Kpna3, Gm8094, Pappa2, Sik2, Gm8127, Gm8212, Dip2b, Hoxa7, Gpr183, Gm8247, Defb30, Rbm47, Tox, Fam168a, Emp2, Magt1, Rbl2, Usp46, 4930539E08Rik, Cask, Rpgrip1l, Slain2, Cep170, Ppp2ca, Dennd2a, Cep350, Sobp, Kif21a, Emp1, Sox6, Fryl, Rfx4, Rnf139, Gsk3b, Zbtb34, Cmc1, Fastk, Gltscr1l, Ago3, Nrk, Tbl1xr1, Papolg, Acvr1c, Npnt, Nid2, Ppfia1, Ahdc1, Map4k3, Dcp2, Prpf39, Dennd1b, Setd5, Top1, Cacna2d1, Khdrbs2, Mgrn1, Ugcg, Asap1, Lrch2, Iffo2, BC005537, Ptpn20, Zfa, Cep135, AA474408, Fbxw7, Mtss1l, Cbfa2t2, Vldlr, Mtpn, Prkce, Senp7, Kbtbd8, Hdgfrp3, Purb, Col8a1, Zfp618, App, Tspan8, Srpk2, Mrgbp, Mecp2, Fubp3, Ptx3, Gm6483, Atp2b2, Kitl, Sh2b3, Etv1, Atp1b4, Smarca4, Gclc, Uri1, Dclre1b, Atp1b2, Rbm41, Sp4, Gch1, Fam188a, Ctdspl2, Stx16, Rasgrf2, Ssu72, Ppp2r5e, Fam98a, Rap1a, Man1c1, Abi1, Hey2, Abhd2, Slitrk6, Mtx3, Npat, Slc35a3, Nxph1, Aebp2, Golga3, Prr11, Oacyl, Crebzf, Naa15, Gspt1, Pcdh19, Sult4a1, AC238840.1, Cdyl, Edem1, Greb1l, Runx1, Mtmr12, Ssfa2, Zfp760, Phlda1, Usp38, Zswim6, Fbxo30, Ank2, Epn2, Cacnb2, Bicd2, Glrb, Zdhhc17, Gpcpd1, Vwa5a, Nckap5, Fam222a, Rsbn1l, Dlg5, Sox1, Als2, Kcnmb2, Rbm12, Mdga2, Arg2, Hnrnpab, Flrt3, Zhx1, Pbx3, Stard8, Tmem161b, Uevld, Gpr50, Ythdf3, Slc16a12, Camkk2, Trmt2a, Itsn2, Ptprj, Dnajc19, Armcx3, Chn2, Insc, Cpeb3, Larp4, Mgea5, Omt2b, Plagl1, Gatsl2, Slc39a10, Lmtk2, Palm2, Wdr17, Plcb1, Gbp6, 6030498E09Rik, Suv420h1, Cxcl10, Fzd8, Tmem65, Msi2, Pcgf5, Gcnt2, Slc18a2, Hdhd2, Rab39b, Sco1, Kcnab1, Sacm1l, Maml2, Stam, Phtf1, Tanc1, Nln, Ptp4a1, Hapln1, Zfp518a, Stc1, Fos, Tbp, Zfp462, Homer1, Prox1, Agfg1, Luc7l, Uqcc1, Zfp874a, Rap2b, Zzz3, Chst11, Serpini1, Tjp1, Fam179b, Klf8, Dram2, Ash1l, Mak, Ctcf, Etnk1, Cav2, Trp53inp2, Cpne3, Map3k9, Col24a1, Zbed6, Tanc2, Ero1l, Prok2, Hnrnpa2b1, Shcbp1, Esp6Esp5, Samd8, Stard13, BC048403, Epha5, Tulp4, Prkaa1, Zswim5, Slc16a4, Tspyl3, Tmco3, Pip5k1b, Dok4, Rab5a, Dnajb14, Nudt12, Slc1a1, Olfr701, Cct4, Lrrn1, Sestd1, BC051076, Tcf4, Mafk, Mtor, Moxd1, Tsga10, Zbtb37, Onecut2, Tgif1, Antxr2, Fem1c, Socs6, Zfp26, Fzd6, Id4, Tceal5, Purg, Ubxn7, Acbd5, Omt2a, Fam206a, Etv5, Zfp37, Sgms2, A630007B06Rik, Rab30, Sub1, Prlr, Akr1c14, Rev3l, Bcl2l11, Nkrf, Trp53inp1, Tmem33, Slc6a11, 1700067P10Rik, Mbtd1, Ick, Rorb |
| miRDB | | Arid1a, Tek, Nfe2l2, Fndc3a, Arid2, Sorcs3, Fat4, Gspt1, Brpf1, Ccng2, Usp42, Ugp2, Slc5a7, Prr11, Gdf10, Uba2, Pfkfb2, Rin2, Zdhhc17, Son, Cpsf6, Pdcl, Arhgap42, Senp7, Pafah1b1, Nacc2, Prickle1, Ets1, Scn1a, Dok4, Ube2g1, Ptpn12, 9930021J03Rik, Fbxl3, Ptpn9, Dtwd1, Rarb, Thap1, E2f8, Meis2, Pcdh18, Kat6a, Hif1a, Eif4g2, Msx1, Fam60a, Impact, Pde7b, Cul5, Atp2b2, Plekhg1, Ahdc1, Pik3c2a, Cask, Tmtc4, Suclg2, Ubxn7, Mcf2l, Atp2b1, Galnt3, Tnfsf11, Fbn2, Pnrc1, Ss18, Ehmt1, Slc12a2, Ireb2, Ube2d3, Slc23a2, Sec24a, Slc8a1, Ptp4a1, Adamts15, Pank1, Hnrnpu, Stard8, Nin, Khdrbs3, Pcsk5, Smad4, Nr2f2, Bach2, Limch1, Zbtb21, Eea1, B230219D22Rik, Cep68, Zbtb18, Sall1, Plat, Ppp1r16b, Crebrf, Herpud1, Sp4, Abi1, Ccdc88a, Gm5531, Slc16a12, Rfx3, Kcnh8, Zfp207, Vps4b, Itsn2, Igip, St18, Fmr1, Itgb1, Gbe1, Mapk1ip1l, Slc25a36, Aldh1a3, Kitl, Pura, Rc3h2, Ms4a4c, Fbxw11, Dlg5, Megf9, Uchl3, Lsm14a, Tmem86b, Rnaseh1, Scfd1, Ccdc85a, Wdfy3, Ptpn4, Mob4, Tjp1, Klf8, Med14, Steap4, Sco1, Tfap4, Osbpl8, Arl6ip6, Strn, Tmtc3, Fam222b, Mapk6, Tnrc6a, Fam196a, Cldn1, Abca1, Zzz3, Ssb, Phtf2, Dcun1d4, Sh3tc2, Tbl1xr1, Zfx, Cct6a, Acsl4, Zranb2, Mmgt1, Hmgcll1, Smoc1, Rbm48, Tet2, Med4, Bclaf1, Fam76b, Antxr2, Vkorc1l1, Meis1, Slco3a1, Trim2, Tmem184c, Cyth3, Pla2g4a, LOC102631639, Hnrnpf, Hat1, Rb1, Fam178a, Tmem65, LOC102631744, Asap2, Acbd5, Mrs2, Ubr3, Cav2, Golt1b, Tada2b, Wif1, Ttn, Magt1, Zfp248, Tspan3 |
| (miR-33-5p) | | |
| TargetScan | | Top2a, Hmga2, Ywhah, Cybrd1, Slc25a25, Hadhb, Pim1, Ik, Gls, Rpp14, Dcun1d5, Chchd7, Rhot2, Rpp14, Foxc1, Dlgap4, Rdh11, Commd8, Styxl1 |
| DIANA | | Gm7293, Abca1, Hmga2, Satb2, Grik2, Setd7, En2, Map4k4, Mlxip, Zfp281, B930036N10Rik, Nufip2, Satb1, Spry1, Hipk2, Slitrk2, Slc12a5, Naa30, Cdk6, Rlim, Sema7a, Mtch2, Naa15, Strbp, Vcan, Cav1, Aard, Rgs7bp, Tph2, Abhd2, Celf2, Ryk, Kcnq5, Map3k7, Tmem14a, Runx1t1, Slc25a25, Igsf10, Asap1, Epha8, Ebf1, Ptgfrn, Fam161a, Ppp1r9a, Esco1, Cntn4, Tanc2, Zfp28, Pigk, Zfhx4, Uty, Eef1a1, Arid5b, Ywhah, Spopl, Tmeff1, Pi4k2a, Prkaa1, Hspa14, Fam126b, Hadhb, Gm5641, Dcun1d1, Rorb, Cep83, Taok1, Ppm1e, Lama3, Glcci1, Gm6793, Cdc42bpa, Rfx3, Braf, Kpna4, Tbc1d5, Clec2h, Gid4, Papola, Slc26a7, Mapk4, Kcnc2, Kcna1, Sos1, Zfp518a, Ncf1, Brwd1, Fut9, Nfia, A930016O22Rik, Tsc22d2, Iqsec2, Skida1, Nipal4, Pdgfra, Rap2a, Tmem86a, Sema3a, Camk2g, Csde1, Morf4l2, Ugt8a, Armcx1, Atxn7l1, Kitl, Cdk16, Nt5dc1, Dmxl1, Srek1, Tet2, Pthlh, Mapk1, Pgrmc2, Zbtb20, S1pr1, Gpr88, Pappa, Hrasls, Scml2, Pcdhb3, Fbxo33, Psd3, Adcyap1, Alg13, Zfp81, Cadm2, Kcnd3, Bcl11a, Hnrnpa3, Il33, Egfem1, Donson, Ttc28, Purb, Btbd3, Nol4l, Scn8a, Vps35, Parpbp, St18, Ddx5, Smc6, Six4, Gabrg3, Nptx1, Brcc3, Dlgap1, Unc79, Msrb3, Nab1, Nrip1 |
| miRDB | | Zfp281, Abca1, Pdgfra, Hadhb, Ywhah, Sec24c, Cacna1c, Cntn4, Crot, Arid5b, Glcci1, Slc25a25, Mdm4, Slc12a5, Vcan, Mtf1, Cntn1, Naa15, Scn8a, Six4, Tmem86a, Gpcpd1, Map3k3, Cntln, Snrk, Fbxw7, Mlxip, Ctnnd1, Tph2, Rgs2, Rora, Abi1, Rgs7bp, Cybrd1, Braf, Txk, 2410002F23Rik, Sema7a, Zc3h12c, Htr5a, Tsku, Setd7, Kcnma1, Dcun1d5, Pim1, Enc1, Sgcb, Naa30, Rap2a, Gpr158, Abcg1, Cadm2 |
| (miR-32-5p) | | |
| TargetScan | | Ugp2, Papd7, Myo1b, Rgs3 |
| DIANA | | Plekhm1, Mycbp2, Ankrd44, Dnajb9, Papd7, Bsdc1, St6gal2, Adam19, Myo1b, Syn2, Tob1, Ube2w, Pnisr, Dcaf6, Mast4, Bcl11a, Golga4, Col27a1, Fnip1, Bcat2, Cpeb3, Tagap1, Cic, Tgif1, Fmn2, Map2k4, Ssfa2, Man2a1, Cd69, Wasl, Sox4, Adam23, Ergic2, Cpeb4, Fbxw7, Exoc5, Usp28, Gramd3, Tagap, Ppp1r37, Hipk3, Edem1, Mia3, Pkdcc, Kat2b, Rad21, Snx13, Bcl2l11, Itga8, Lhfpl2, Chmp7, Klf4, Bcl11b, Atxn3, Nefl, Runx1t1, Cpeb2, Slc12a5, Tob2, Evx2, Evi5, Ccnjl, Mfhas1, Necap1, Ugp2, Gpr180, Dus2, Fmr1, Snn, Lmbr1l, Rev3l, Baz2b, Avl9, Gramd1b, Adamtsl3, Tbx20, Rnf141, Map1b, Pcdh11x, Pgam1, Itgav, Cacna1i, Arl5b, Btg2, Fam126b, Gfpt2, Pcolce2, Nox4, Srpr, Klf2, Fam135a, Nckap5, Fzd10, Cep41, Cldn11, Ptar1, Foxn2, Rgs17, Spryd4, Phlpp2, Arid1b, Atrx, Arrdc3, Ppp1r12c, Slc24a3, Dkk3, Sorl1, Mylip, Kcnc4, Itpr1, Fam19a1, Pde10a, Tef, Morc3, Atp8b1, Sim2, Dusp10, Tram2, Rhpn2, Sik1, Wwp2, Nkx2-3, Rnf38, Abhd13, Arhgef17, Lcor, Itga5, Dennd1b, Rsbn1, Scn8a, Rab8b, A830018L16Rik, Slx4, Cbln4, A830010M20Rik, Gdf11, Usp36, Ibtk, Kntc1, Rpl15, Smad7, Lurap1l, Tsc1, Adamtsl1, Dab2ip, Grhl1, Wrnip1, Stx17, Col1a2, Cdk16, Gpbp1l1, C030046E11Rik, Ptpro, Pdzd2, Glra1, Sun1, Dnajc4, BC005537, Josd1, Wdfy3, Gata2, Tmem229a, Dnajb12, Ibsp, Cbfa2t3, Asxl2, Fnip2, Rest, Fst, Ddx3x, Pramel7, Fam160b1, Gpr137c, Per2, Pik3cb, Rbm47, B230219D22Rik, Fbn1, Fndc3b, Strn3, Phtf2, Luzp1, Trim36, Rnf44, Rnf4, Gpr98, Rbm27, Gltscr1l, Rap1b, Ppcs, Tcf21, Gid4, Sfxn1, Nlk, Pitpnm2, Syne1, Otud4, Rab9b, Prdm13, Mtdh, Glyr1, Synj1, Eif4g2, Ptprj, Tub, Ptpre, Hand2, Sgk1, Adam10, Rora, Zfp521, Zeb2, Cog3, Mier3, Dpy30, Mark1, Cttnbp2, Iqgap2, Polk, Dmxl1, Pten, Unc79, Csmd3, Nfyc, Plekha1, Gnpda2, Fam110b, Jade1, Zdhhc5, Ahcyl1, Armc1, Prkar1b, Zbtb20, Kctd18, Braf, Cyp2d22, Golga1, Nsf, Slc25a32, Dusp5, Efr3a, Gm28040, Hivep1, Dock9, Snap29, Robo2, Pcmtd1, Zim1, Arpc2, Gm10639, Rhox2g, Osbpl8, Hecw1, Socs5, G3bp2, Sfmbt2, Greb1l, Sertad2, Ptprg, Papola, Osbpl9, Elovl4, Isca1, Klhl29, Fancm, Zfp597, Zfp827, Cd200r1, Nutf2, Chd9, Sgpp1, Rbfox2, Kcna1, Jmy, Dcp1a, Zdhhc15, AK157302, Klhl15, Pcdh10, Tead1, Hoxd10, Arrdc4, Sec24a, B3galt2, Xylt2, Ikzf4, Pds5b, Ube2z, Gla, Znf512b, Snapc1, Nsmaf, Rbpj, Hps6, Ikzf1, Appl1, Gpr158, Cadm2, Stau1, Zfyve21, Aars, Prdm16, Setd5, Bgn, Rab3c, E2f3, C530008M17Rik, Ttc9, Mef2d, Pik3r3, Slc17a6, Tbc1d12, Hcn2, Pip5k1c, Cdkn1c, Cacna1c, Gsta2, Sort1, Upf2, Ago3, Cdc42bpa, Skor1, Tacc2, Cdh8, Dsc2, Peak1, Ythdc1, Nfia, Dcbld1, Frmd7, Gca, Pax3, Ppp1r9a, Tpcn1, Tbl1xr1, Zfc3h1, Sema3a, Ptprd, Fam160a2, Sgk3, Angptl2, Eif1, Tnpo1, Sh3pxd2a, Zak, Gm14698, Elk4, Mpp1, Zfp804a, Ptprk, Zbtb18, Ptger4, Myh3, Atrnl1, Lin54, Hand1, Glce, Pof1b, Zbtb34, Rps20, Hook1, Cxxc5, Sbno1, Jade2, Papd5, Fcho2, Golga3, Nefh, Zfp654, Lmo2, Nol7, Gmps, Pepd, Lrch1, Satb2, Rab23, Dmrtc1c1, Fbxo33, Herc2, Gm4980, Megf10, Pcgf3, Rabggtb, Slc12a2, Rab14, Prkar1a, BC061212, Gtf2a1, Rilp, Oser1, Atxn1, Bahcc1, Nfib, Usf2, Col11a1, Mdh1, Pmepa1, Hnrnpu, Corin, Zfhx3, Alx4, B630019K06Rik, Snrk, Dclk2, Rbpms2, Cux1, Cnot2, Gan, Erbb2ip, Neurl1b, En2, Pp2d1, Slc6a1, Trhde, Xrn1, Clec16a, Herpud2, Rps6kb1, Krtap16-1, Nova1, Fkbp1a, Dag1, Dmrtc1a, Twist1, Socs6, Eomes, Dmrtc1c2, Paip1, Tnrc6b, Fam214a, Col12a1, Tcf4, Fam196b, Zfp280d, Zcchc2, Dynlt3, Syndig1, Usp33, Ddc, Sestd1, Myt1l, Gm5148, Kcnk3, Prdx5, Mapk8, Wwox, Ncoa3, Meis1, Klhl14, Pdzd8, Arf1, Snx25, Foxp2, Slitrk5, Cend1, Aggf1, Tulp4, Tenm1, Csmd1, Grk5, Pvrl1, Cacna2d1, P2ry13, Sar1b, Mitf, Dpp10, Tmcc1, Kcnh7, Abi3bp, Stk16, Hoxc8, Col5a1, Chga, Sdr9c7, Desi1, S1pr1, Cpne8, Zkscan17, Golga7, Vstm5, Smurf1, Gpr173, Foxp1, Daam1, Fbxo28, Caln1, Pitpna, Phf3, Fam76b, Myo5a, Fli1, Rabgap1l, Nek1, Wwc1, Myo1c, Notch1, Lgalsl, Alpk3, Slitrk4, Cdca7l, Ptpn14, Mtf2, Bcl9, N4bp2, Tmem184b, Ndufa5, Krt1, Map4k5, Ext1, Tbc1d19, Akap1, Ctnnb1, Lats1, Rnf214, Clip4, Atp11c, Ttc39b, Pfkfb4, Zfp672, Rhox2d, Tet2, Rab3ip, Baz2a, 4932438A13Rik, Bmp2k, 5430421N21Rik, Robo1, Atg14, Stag2, Lifr, Gclm, Ikzf2, Negr1, Nrg4, Galnt14, Ddx3y, Prkaa2, D630003M21Rik, Mmp16, Trp63, Cd2ap, Hnf1b, Slc28a3, Grp, Phldb2, Rrbp1, Zfp532 |
| miRDB | | Dnajb9, Dsc2, Rsbn1, Fbxw7, Slc12a5, Ergic2, Cpeb3, Zfyve21, Morc3, Ppcs, Dcaf6, Tob1, C030046E11Rik, Zfc3h1, Wasl, Usp28, Cd69, Rhpn2, Hivep1, Slx4, Xrn1, Dynlt3, Isca1, Map2k4, Nefm, Ptar1, Fam160b1, Itpr1, Hipk3, Snx13, Bsdc1, Chka, 4932438A13Rik, Grhl1, Pcdh11x, Man2a1, Myo1b, Suv420h1, Tef, Cep41, Gramd3, Efr3a, Gfpt2, Ube2z, G3bp2, Pp2d1, Itgav, Adamtsl3, Ccnjl, Slc24a3, Spryd4, Ankib1, Tob2, Btg2, Srpr, Lhfpl2, Ssfa2, Adam10, Pcolce2, Ugp2, Nckap5, Map1b, Foxn2, Ddc, Ccnc, Zeb2, Ptpro, Fnip1, Bcl11b, Cldn11, Myo5a, Kat2b, Pcgf3, Peak1, Papd7, Hps6, Cdh10, Mtf1, Nova1, Fmr1, Wrnip1, Ppp1r37, Rgs3, Slc25a32, Klf4, Lats2, Tbl1xr1, Sgpp1, Nox4, Atxn3, Cic, Rbpms2, Fam19a1, Ankrd44, Hand2, Hnf1b, Plekha1, Dpy30, Gpr180, Dpp10, Prkar1a, Herpud2, Synj1, Arid1b, Appl1, Aars, Wwp2, Aida, Cog3, Fhl2, Pcmtd1, Necap1, Avl9, Per2, Yipf4, Ddx3x, Mia3, Snn, Mapk8, Leprel2, Klhl29, Pkdcc, Adamtsl1, Sim2, Gpbp1l1, Nlk, Phlpp2, Nsmf, Eomes, Dscaml1, Fam20c, Cdca7l, Bcl11a, Pcdh7, Syn2, Sertad3, Exoc5, Tagap, Med19, Arrdc3, Itga6, Fam135a, Mycbp2, Tgif1, Scn8a, Glyr1, Ubash3b, Dnajc30, Rgs17, Dkk3, Trio, Gm10639, Ptger4, Osbpl8, Cnep1r1, Elovl4, Klhl14, Fry, Col1a2, Kcnk10, Sfrs18, Marf1, Dmxl1, Rad21, C3ar1, 1810055G02Rik, Tfdp2, Gsta2, Fbn1, Iqgap2, Tmem229a, Sdc2, Prkar1b, Mfsd7c, Gpc6, Slc25a36, Rab14, Rev3l, Socs5, Atp7a, Pof1b, Rassf3, Tulp4, Ptprj, Golga4, Luzp1, Gata2, Pitpnm2, Anp32e, Gpr98, Phtf2, Slco6c1 |
| (miR-1983) | | |
| TargetScan | | Clu, Tmem106c, Hnrnpa2b1, Oaz1, Myc, Aldoa, Cd59, Nhp2l1, Slc16a3, Hn1, Ptprf, Xrcc5, Itgb1bp1, Rnf187, F3, Ssr1, Hnrnpul1, Ube2d2, Col6a3, Igfbp5, Cltc, Hdgf, Ctgf, Ccar2, Tspan3, Ppif, Qsox1, Srsf2, Nap1l1, Pls3, Sh3bgrl3, Mrpl11, Atp5g3, Bcl2l1, Chid1, Ywhah, Atp2b4, Ptbp1, Cdk2, Fstl1, Mcts1, Msrb2, Pdxdc1, Prmt7, Pdcd6ip, Rnf11, Atic, Actn4, Pdxk, Ube2g1, Fxyd5, Pdk4, Ndufb5, Vcp, Mrpl22, Rpl15, Mtdh, Chpf, Etfa, Ccnd1, Cast, Klf6, Acss2, Nr2f2, Palld, Emc3, Avpi1, Col5a2, Sh3bp5, Dazap2, Gltpd1, Trnp1, Clpp, Rbm14, Agfg2, Calm3, Hdac3, Shisa5, Map4, Cbfb, C2orf72, Prpf31, Ubxn4, Psme3, Cstf2t, Trim28, Nek6, Myadm, Ube2b, Lancl1, Plekha2, Vps37c, Chmp1b, Sf3b1, Sec63, Crtap, Timp2, Fam50a, Ubl4a, Mrps35, Znf146, Mrps12, Znf282, Tceb3, Ube2j2, Ankrd32, Srpr, Luc7l3, Sec61a1, Mea1, Podxl, Vapa, Impa2, Shroom3, Edil3, Adamts2, Aff4, Gde1, Prpf4, Fam104a, B3gnt1, Itpkc, Hdgfrp3, Kbtbd2, Sf3b3, Wls, Aagab, Acsl3, Trabd, Zfand5, Asxl1, Tor1a, Kat8, Cyb5b, Galnt1, Lman2, Usp9x, Itgav, Snapc3, Lhpp, Pcsk9, Mgrn1, Hnrnpul2, Furin, Lman1, Vkorc1l1, Fosl2, Ndufa10, Bag2, Rai14, Pgam5, Tmem237, Ndrg1, Npepps, Mmp2, Cnih1, Glg1, Tmem8a, Keap1, Crkl, Tmem123, Hmgn3, Pomgnt1, Klhl42, Pex5, Sort1, Gpx8, Pgrmc2, Wnt5a, Tmem37, Efemp2, Tnpo1, Sdf4, Ercc1, Agfg1, Ndufc2, Ssbp2, Hddc2, C6orf89, Fah, Slc38a1, Afap1, Ppt1, Hif1a, Zmat2, Srsf10, Srsf6, Lpp, Gpank1, Hspa4, Rtf1, Crat, Slc25a11, Dut, Sept6, Crk, Rab21, Atg12, Gapvd1, Pex19, Yy1, Dbnl, Sgcb, Oxct1, C11orf58, Sco1, Insig1, Cpd, Hnrnpd, Dgcr2, Tmed10, Ndfip2, Mical2, Mmp11, Sod1, Gpr161, Mbtps2, Hdac2, Btg1, Exosc3, Mcfd2, Spcs3, Tmem158, Mgat5, Rnf44, Dld, Marc1, Epn2, Nfe2l1, St20-Mthfs, Mthfs, Gtf3c4, Aacs, Mpc2, C9orf3, Bhlhe40, Cand1, Tnfaip1, Map4k4, Hif1an, Cd44, Ylpm1, Rbm23, Tp53bp2, Man2a1, Gna11, Desi1, Rnf146, Pds5b, Pi4k2a, Pi4k2a, Dcun1d5, Ccdc47, Drg1, Atg7, Rpl22, Psmg4, Mrps11, Oaf, Sept2, Tet3, Shb, Gpr107, Vta1, Ets1, Ran, Ubp1, C6orf120, Cnot3, Msmo1, Mcmbp, Rab18, Tspan9, Sar1b, Hmgcr, Bcl2l2, Igf2, Rpa3, C1orf112, Celsr1, Amfr, Psen1, Arhgap17, Ube3c, Vps53, Gnl1, Rbm3, Tmem245, Cpa4, C12orf52, Zkscan1, Arrdc3, Gtf2b, Snx1, Dpp8, Rassf8, Dph3, Ahcyl1, Fam122a, Txnrd1, Bnip2, Btf3l4, Cuedc1, Leprotl1, Chd2, Slc7a2, Wdr26, Aebp2, Wdr77, Eif2b2, Prelid1, Cmc1, Smad2, Ebf4, Pycr2, Cnot2, Dlgap4, Pde3a, Txndc15, Plekha1, Gng4, Tpd52l1, Dynll2, Camkk2, Snx19, Fmr1, Nfib, Zbtb9, Tmem185b, Chst14, Pbx3, Rprd1b, Itgb3bp, Sprtn, Vav3, Prrg1, Mrps21, Ctb-102l5.4, Ralgapb, Dcun1d4, Cdk6, Stc2, Frmd8, Smim12, Dnajc5, Fgfr3, Stx12, Oser1, Atg5, Cited2, Manba, Rragc, Tsc22d2, Sox13, Xxylt1, Rfx7, Slc7a6, Rps6ka2, Gpr125, Zbtb4, Wipi2, Nxf1, Hspa14, Pigs, Rnf167, Tek |
| DIANA | | Hipk3, A230083G16Rik, Tcf20, Slc5a12, Itga6, Prb1, Gm8882, Lpin2, Rab8b, Clock, Mapk9, Ephb1, Etos1, Stc2, Mapk10, 9130019O22Rik, Ppm1h, Xab2, Srsf2, Fancd2, Ifnlr1, Tfpt, Sh3gl3, Nol4l, Rsf1, Abca5, BB014433, Gm9913, Ccdc92, Flot2, Flna, Pilrb2, Zfp810, Zfp157, Gsx2, Bach1, Acbd4, Pcsk2, Zfp866, Ankrd13d, Mill1, Crebzf, Gdf11, Zfp68, Ccdc178, Ccdc117, 4930571K23Rik, Slc35d1, Pla2g4b, Spty2d1, Egr2, Gltpd1, Dennd2a, Rbpj, Rnf44, Lig3, Frmd4a, Tmem132a, Gtpbp2, Lonrf2, Ctu1, 2810021J22Rik, Katnbl1, Mdga2, Rnf144a, Itpr2, Hnrnpll, Rps20, Zmynd11, Zfp820 |
| miRDB | | Tmem178b, Srsf2, Xkr7, Unc119b, Snx12, Rims2, Zmynd11, Clock, Cmtm4, Zfp516, Tmem230, Otud7b, Nfat5, Ell, Igsf9, Nrsn1, Rab8b, Sv2b, Hmox2, Fxn, Fzd3, Mknk2, Phlpp1, Arrdc3, Hipk3, Ube2g1, Rab39b, Fam134c, Zfp148, Hnrnpll, Crkl, BC048403, Mical3, Spty2d1, Shank2, Hnf4g, Tdrkh, Nufip1, Ppm1h, Seh1l, Bach2, Pcsk2, Prpmp5, Zfp764, Bach1, Hip1, Degs2, Lrig2, Gpr55, Nfatc3, Pik3cd, Klra8, Ehd4, P2rx7 |
| (miR-136-5p) | | |
| TargetScan | | Clpp, Rexo2, Cbx4, Nnt, Pop7, Ccng1, Rasl10b, Psmg4, Mtpn, Luzp6, Fam122a |
| DIANA | | 4932414N04Rik, Bptf, Wdr43, Sema4c, Braf, Mtpn, Ppp1r18, Mrps16, Extl3, Rpgrip1l, Etf1, 1700019G17Rik, Cbx4, Slc26a3, Zfp827, Ankrd42, Purb, Serf2, Ino80, Htatip2, Dcaf7, Chrdl1, Blmh, Eif4g3, Ccdc14, Cntn1, Gria1, Stt3b, Dab2ip, Mab21l1, Eif2a, Fmr1, Olfr701, Rin2, Tnfsf8, B230219D22Rik, Rpusd4, Ccdc150, Dixdc1, Esrrg, Cpeb2, Ncoa6, Usp32, Dis3, Crebzf, Gas2l3, Serinc3, Pdpn, Zfp710, Rap2c, Ppargc1a, Sdpr, Taf7, Zic3, Rad50, Homer1, Kcnd1, Tuba3b, 5031425E22Rik, Rnf139, Msl2, Psmd13, Cdv3, Map2k4, C1qtnf7, Iqck, Poc1b, Atrn, Hoxc10, Ndrg4, Oasl1, Cfh, Fam120a, Cntrob, Ppp2r2a, Gm13821, Bnip3l, Sf1, Jazf1, Lcorl, Sumo2, Dennd1a, Lrrc3, Nono, Rgs4, Pdgfra, Rab21, Ros1, Aym1, Sncaip, Katnal2, Tmc1, C2cd5, Hectd3, Nfib, Ube2g1, Frrs1, Sept3, Arhgef9, Ppfia2, Fam65b, Impg2, 2900092C05Rik, Gdi2, Gm5155, Ntng1, Eapp, Lypd1, Arf6, Trip10, Kalrn, Fam160a2, Kctd3, Ggct, Mtch2, Acin1, Zfp451, Yes1, Tekt2, Lrrc58, Pura, Tmem233, Rnf115, Hells, Mllt3, Ylpm1, Ngrn, Pbx3, Mfap3l, Magt1, Phactr3, 5830415F09Rik, Zfp532, Wfdc5, Pprc1, Gm10419, Cntn2, Hoxa9, Gm10742, Ddn, Nr1h5 |
| miRDB | | Slc7a3, Mtmr4, Rpusd4, Radil, Arpp21, Atrn, Ino80, Tmem178b, Cnot7, Prpf38b, Rasl10b, Rif1, Braf, Gria1, Mtmr7, Jazf1, Sec14l4, Trpc4ap, Tnrc18, Wdr43, AI317395, Crebzf, Ccng1, Ppp2r2a, Mllt3, Zmym2, Ankrd12, Fmr1, Cntn2, Snrk, Ate1, Irak2, Tmem161b, Sema4c, Slc35a5, Oaf, Epha4, Pwwp2a, Sytl4, Arf6, Sgip1, Rnf139 |
| (miR-142-3p) | | |
| TargetScan | | None |
| DIANA | | Suco, Utrn, Map3k11, Prlr, Mbd6, Rab2a, Rock2, Tab2, Cask, Huwe1, 4930402H24Rik, Arntl, Rac1, Zfp217, Bach2, Foxo4, Mlxip, Cpeb2, Pum1, Zeb2, Tex43, Wasl, Zbtb20, Tgfbr1, Aff2, Ash1l, Hectd1, Itgav, 2010106E10Rik, Zfp827, Inpp5a, Sp4, Tceb3, Rlf, Slc37a3, Twf1, Apc, Tnks, Strn3, 2210010C04Rik, Trpc5, Sh2b1, Rere, Irak1, Lrrc59, Fam114a1, Ehf, Ptbp3, Il6st, Atg16l1, Dirc2, Fyco1, Stx12, Rab12, Rgl2, Taok1, Brwd3, Morf4l2, Trpc3, Kat2b, Ptpn23, Dcun1d4, Egfl6, Zfp36l2, Stau1, AW549877, Fam208b, Wdr62, Rictor, Pde4b, Slc1a3, Ddhd1, Ict1, Baz1a, Gas2l3, Mgat4a, Myh10, Marcks, Spag17, Lcor, Lpcat3, Gnaq, Zfp708, Il7, Grm7, Bod1, Gorab, Rnf219, Cops7a, Tmem56, Tfg, 1700055D18Rik, Nkx2-3, Ppp1r37, Kdelr2, Cmah, Mier3, Thap2, Prpf39, Npsr1, Nr3c1, Zeb1, ECd200r4, Esyt3, Rarg, Rab3a, Acbd5, Stag1, Ccdc132, Rbm27, Bnc2, 1810046K07Rik, Pagr1b, Fmn1, Rreb1, Ier3, Sik1, Sgk1, Gnb2, Il17d, Trak1, I830077J02Rik, Gm8975, Eml4, Ank3, Golga1, Meis1, Tyw5, Cfl2, Lrp1b, Fndc3a, Fkbp1a, Tbl1xr1, Gfi1, Hgs, Tmem55b, 3110043O21Rik, Leprel1, Tardbp, Dsel, D1Pas1, Cxadr, Ttc26, Cul4b, Pmaip1, Ctsm, Kif5b, Acsl4, Zfp654, Mospd1, Atp8b1, Arl4a, Zbtb41, Akirin2, Itgb8, Dhtkd1, Slc35f6, Sfi1, Tmem110, Picalm, Vsx1, Klf13, Egr2, Lats1, Dcakd, Bcl2l1, Itpr3, Dcaf12l1, Arhgap35, Sik2, Rab11fip2, Zcchc14, Cd151, Atxn7, Crk, Tbc1d2b, Slc20a2, Usp21, Psmd11, Xpo1, Nol4l, Dmtf1, Gm14692, Sp8, 9230019H11Rik, Dbx2, Hmga2, Gm11596, Arhgap42, Snx18, Foxn1, Tnfrsf13c, Kdm6a, Hykk, Mc4r, Hist1h2bq, Il1rapl1, Fabp4, Atg4c, Vps8, Nudt12, Slc39a10, Sox6, Rab14, Fam196b, Asrgl1, Rab1 |
| miRDB | | Hectd1, Kat2b, Rlf, Eml4, Rictor, Wasl, Fmnl2, Rab23, Itgav, Sik1, Dirc2, Kif5b, Foxo4, Egfl6, Morf4l2, Lrrc1, Zbtb41, Fyco1, Strn3, Tgfbr1, Baz1a, Tmem59, Stam, Utrn, Akt1s1, Arntl, Ptpn23, Rheb, Inpp5a, Tnrc18, Brwd3, Rgl2, Twf1, Zeb2, Rimklb, Reps2, Atxn1l, Myh10, Tsen34, Cxadr, Nap1l5, Trpc3, Kat7, Vamp5, Fbxo3, Bach1, BC016423, Rab39, Braf, Mlxip, Taok1, Apc, Fam46a, Marcks, Mbd6 |
| (miR-376a-3p) | | |
| TargetScan | | Myc, Itgb1bp1, Gabarap, Mt-Nd4l, Fstl1, Mcts1, Wrap73, Prmt7, Scarb2, Slc39a7, Tnc, Nid2, Tsc22d1, Lancl1, Cap2, Stc1, Fyn, Eps8, Marveld1, Oaz2, Dkk1, Lman1, Cnbp, Actr10, Cachd1, Atp2a2, Pawr, Crk, Dbnl, Dgcr2, Dld, Pigp, Ltbp2, Pafah1b1, Tnfaip1, Mark3, Uggt1, Arhgap17, Bmp2, Fads1, Mboat2, Stc2, Reep3, Kmt2a |
| DIANA | | Kmt2a, Stc1, Gmnc, Gal3st4, Bmp2 |
| miRDB | | Entpd1, Atp2a2 |
| (miR-142-5p) or (miR-142a-5p) | | |
| TargetScan | | Cyfip1, Rnh1, Rhoc, Rhoa, Hnrnph3, Emc4, Mrps6, Btg3, Znf146, Mal2, Rnf146, Btf3l4, Ccng2, Smim15 |
| DIANA | | Lrp1b, Bai3, Zbtb20, Zfpm2, Atp13a3, Rnf165, Rev3l, Otud4, Cnot6, Elavl4, Lmx1a, Tnfrsf26, Robo1, Zcchc14, Herpud1, Setd2, Abca1, Taf3, Slc4a4, Ccng2, Prpf40a, Fign, Elavl2, Pura, Cdk13, Atxn7l2, Son, Hipk1, Wwp1, Kitl, Btg3, Nampt, D15Ertd621e, Rsf1, Tns1, Msl2, Dock4, Lpp, Fam160a2, Klhl14, Necab1, Tcf12, Ccdc6, Sel1l3, Vmp1, Rhot1, Sox5, Rnh1, Ankib1, Qk, Mycn, Cpeb2, N4bp2l2, Adamts1, Pds5b, Med12l, Fgf15, Zc3h12c, Mical2, AK129341, Tcf4, Pcdhb3, Med14, Bicd1, C77370, Ppp2r5c, Mylip, Smurf2, Arid1a, A830018L16Rik, Vps54, Vmn1r51, Maml1, Pgr15l, Brwd3, Tmem245, Six4, Fam134a, Pi15, Lrrc58, Cntn1, Lhx9, Capn7, Tbl1xr1, Slc23a2, Man1a, Col4a3bp, Cask, Ddx5, Lsamp, Il21, Ndfip2, Eif2a, Cpsf6, Macf1, Gdnf, Zfp275, Lrp2, Rbm24, Uba3, Cadm1, Asxl2, Ube2a, Wdr16, Spock2, Mastl, Ube2d1, Otx2, AI467606, Psd3, Btf3l4, Cdc37l1, Epas1, Phlda1, Ptbp3, Iltifb, Kif13a, Rhobtb3, Rnf150, Arap2, Cep170, Vezt, Pou3f1, Kif5c, Npas4, Abi2, Slitrk4, Rab6b, Plekha3, Frmd5, Nucks1, Fbxo33, Myt1l, Mal2, B4galt6, Gpc6, Rgs17, Dgke, Ldb2, Ncapg2, Slc18a2, Armc8, Npat, Ccdc117, Tsc22d2, Zfp462, Als2, Dlg1, Pcgf3, Arid2, Mbnl1, Magi2, Stx19, Camsap2, Chd9, Pappa, Fam199x, Igf1, Dab1, Etv1, Ghr, Srsf6, Gmfb, Dazl, Dlg2, Ccdc47, Tfap2b, Tiam1, Nanos1, Zic3, Rbbp8, Rc3h1, Jmjd1c, Prrg1, Rps6ka4, Pank3, Stau1, Bcor, Ppp1r42, Atrx, Fgf13, Scin, Tmem54, Hn1, Lrch2, Stt3b, Ubl3, Pde1c, Klf10, Slain1, Slfn4, Cbx3, Rybp, Cbln4, Shisa6, Mbip, Ddx6, Ankrd17, Igf2bp3, Acin1, Zfp148, Tmod2, D930020B18Rik, Ppp4r2, Zfx, Nkx3-1, Gabpb2, Ift80, Ppfia2, Hiat1, Usp9x, Naa30, Dclk1, Kit, Commd2, Egln3, CG1273, Cdk17, Prss8, Camk4, Bmp2, Arhgef12, Mbd2, Hoxa10, Fam126a, Tbc1d8b, C1d, Ywhaz, Fyttd1, Hook3, Fam168a, Scoc, Emb, Msrb3, S1pr1, Gulp1, H3f3a, Pclo, Prdm4, Rhoa, Tgfbr2, Dsp, Acvr2a, Pcdh11x, Cttnbp2, Dram2, Ptpn4, Zdhhc20, 1110037F02Rik, Fgf4, Etaa1, Lins, Stk35, Sertad4, Gpr174, Bnip2, Cep97, Adamts5, Atl2, Rab11fip5, A830010M20Rik, Alcam, Dcdc2a, Pphln1, Stc1, Ext1, Aff4, Osbpl8, Pum1, Lmbrd1, Hax1, Rap1a, Itgb8, Ankrd28, Ginm1, Slc17a7, Hoxc8, Brinp3, Krt222, Slc25a37, Eda2r, Unc80, Bmp2k, Mcl1, Rasa1, Nr1d2, Tbc1d9, Vangl1, Tmtc1, Ing3, Lcorl, Clasp2, Mob1a, Efcab14, Ago3, Cblb, Acadsb, Hoxd8, Vrk1, Sp4, Fkbp7, Vmn1r4, Map4k3, Fam13c, Tm6sf1, Tmem67, Zic5, Rhobtb1, Prpf4b, Tmem158, Slc24a2, Dusp2, Sult1d1, Armcx4, Phf6, Zfp503, Nrxn1, Nkiras1, Dio2, Atxn7, Trip11, Bmpr1a, Ddx26b, Npas3, Rag1, Pcdh19, Tdrd3, Hltf, Dip2c, Ssh2, Dnal1, Fam120a, Fbxl3, Nck2, Rhoq, Dlgap1, Klhl15, G2e3, Id2, Sra1, Jarid2, Mdh1, Atp1b1, BC024978, Tmem68, Lphn3, Ppip5k2, Mtdh, Osgep, Acer2, Zdhhc17, Fam19a1, Zbtb44, Rprd1a, Spopl, Cd274, Pcdh18, Usp48, 3830403N18Rik, Rgmb, Eya4, Zfp616, 1700006A11Rik, Trpm7, Tspan14, Synj1, Csnk1g3, Cpeb3, Dnajc7, Wdr47, Gk5, Rerg, Megf10, Bcl11a, Abca8b, Tg, Hist1h1d, Diap2, Carf, Zfp36, Sostdc1, Cpd, Scfd2, Fndc3b, Vstm2a, Uhmk1, Slc25a27, Rab12, Slc25a40, Klf11, Casp12, Sppl2a, Ranbp1, Zfp451, Cadm2, Slco6c1, Set, Lmbrd2, Grsf1, Trp63, Sema3e, Foxa1, Sgce, Med28, Gm4297, Smad7, Zfp664, Nfat5, Scml2, Hp1bp3, Trdmt1, Fgg, Pcdh15, Ccdc88a, Axin2, Suco, Pdcd6ip, Zfp654, Gas7, Nfia, Hipk2, Zbtb10, Prune2, Jrkl, Mospd2, Robo2, BC030336, Gpr119, 2410002F23Rik, Unc5d, Arpc5, Rimklb, Gmcl1, Cacnb4, Fgl2, Impad1, Atf7ip, 2410131K14Rik, Tnfsf8, Dnajc25, Tmx3, Caprin2, Slc39a8, Lrp8, Dmd, Gpr21, Map3k2, Uty, Csrnp3, Fam126b, Rhoc, Slc4a7, Uchl3, Crim1, Rbm47, Gpr75, Rlim, Ubr1, Dpp10, Gm6984, Ctdspl2, Map3k13, Diap3, Usp31, Lrat, Soga1, Gm2030, Dcun1d4, Myo1d, Nedd1, Gpd2, Dnajc3, Phip, St8sia2, Marcks, Rab3gap2, Nhsl2, Acvr1c, Slc15a5, Kif15, Eogt, Iws1, Ryk, Aard, Itga8, Mageb16, Gm3099, Gopc, Slco1a5, Pgm3, Bhlhe22, Stag1, Dusp11, Mbd3l1, Rb1cc1, Sept2, Srebf1, Zfp618, Cuedc2, Papd5, Vmn1r132, Magi1, Fbxo21, Rab22a, Hecw2, Gclm, Sde2, Pbx3, Cdon |
| miRDB | | Lmx1a, Zfpm2, Vmp1, Bai3, Atxn7l2, Kif5c, Med14, Lrp2, Gpr75, Igf2bp3, Zfp275, Dynlt1b, Srsf6, Adamts1, Arid2, Ptpn4, Ube2a, A830018L16Rik, Robo1, Actn4, Commd2, Slc16a9, Elavl4, Slain1, Stau1, Efcab14, Garem, Chsy3, Bicd1, Vps54, Stc1, Nck2, Lhfpl3, Synj1, Fam19a1, Dynlt1f, Dynlt1c, Ube2d1, Ckap2l, Hnrnph3, Camta1, Capn7, Pp2d1, Diap2, 1700010I14Rik, Etv1, Cdk17, Kif13a, Setd2, Mtmr10, Hipk1, Zcchc11, Pcgf3, Kras, Rap1a, Stag1, Kitl, Zfp770, Magt1, Rab6b, Klf11, Rhoq, Cnep1r1, Plxna2, Nedd1, Zc3h12c, Uba3, Spire1, Gtf2a1, Ivns1abp, Pds5b, Iltifb, Med28, Sh3rf3, Aff4, Lrp12, Prpf4b, Gdnf, Prkg1, Rnf165, Dnajc25, Ccng2, Btbd7, Chrna5, Cdc42ep4, Mal2, Caprin2, Rsf1, Cisd2, Mthfr, Zfp651, Rev3l, Fam126b, Dynlt1a, Gas7, Zmym2, Ube4a, Prpf4, Slc18a2, Cbln4, Npat, Tdo2, Cul4a, Arap2, Wwp1, Gopc, Gngt1, Hmgcll1, BC030307, Pigw, Klf10, Hook3, Olfr630, Depdc1a, Btf3l4, Dio2, Myo1d, Grm7, Etfdh, Grsf1, Necab1, Herpud1, Hdlbp, Brd3, Fam131b, Cul2, Macrod2, Cntn1, Fam199x, Tiam1, Bmpr2, Fam19a2, Tmem245, Lhx8, Slc1a2, Sorbs1, Prkaa2, Mbd2, Slc12a1, Fbxl3, AI467606, Becn1, Cbx3, Rhoc, Mgat4b, Prex1, Sgce, Atg16l1, Spin4, Mtmr2, Nav3, Cox16, Camsap2, Cnnm4, C77370, Map3k13, Zbtb37, Tmx3, Rnf146, Fam134b, Prpf40a, Zfp503, Arhgef12 |
| (miR-3968) | | |
| TargetScan | | Col1a1, Tmem106c, Prdx6, Myc, Ddb1, Sars, Nhp2l1, Sf1, Rbp4, Slc16a3, Xrcc5, Jund, Ndst1, Rnf187, Mlec, Klc2, **Stoml2**, Prss23, Tpm2, Arglu1, Peg10, Irf2bp2, Rpl14, Atp6v1g1, Ccdc124, Jun, Atp2b4, Ptbp1, Ccnb1, Cdk2, Camk2n1, Col4a1, C14orf119, Wrap73, Sox12, Prmt7, Ssr3, Melk, Rab11a, Ptp4a1, Dkk3, Actr1b, Hnrnpu, Bub3, Mtdh, Tbc1d9b, Pdcd6, Mthfd1, Cast, Hs6st2, Grsf1, G3bp2, Trim44, Igfbp4, Map4, Phlda1, C2orf72, Alkbh7, Pip5k1a, Ezr, Epas1, Lamtor5, Eif2s1, Acvr1b, Lrp11, Psph, Znf593, Lamp2, Dpysl2, Ulk1, Myo1b, Mtss1l, Iars2, Luc7l3, Stc1, Vapa, Nptn, F11r, Rpa1, Elavl1, Mcm6, Tram2, Adamts2, Sox4, Psmf1, Atf3, Arhgef18, Sox9, Srsf3, Tmem43, Psma2, Triap1, Tspan7, Asxl1, Kctd15, Fibp, Cyb5b, Ddit4, Usp9x, Nipsnap1, Sypl1, Ykt6, Mgrn1, Mthfd1l, Adsl, Odf2, Anxa11, B4galt1, Fosl2, Ddx17, Tmbim1, Cnih1, Glg1, Tmem8a, Hmgn3, Pex5, Lpar1, Chp1, Sbf1, Dera, Fubp1, Spryd4, Ssbp2, Nbr1, C6orf89, Slc38a1, Ppt1, Hif1a, Phyh, Csnk2a1, Parva, Clstn1, Lpp, Ncapd2, Pawr, Crk, Dbnl, Nceh1, Gtpbp1, Hnrnpd, Ppp2r5d, Bloc1s6, Txndc17, Emp2, Rasa1, Senp2, Gpc1, Btg1, Vasp, Mgat5, Smc1a, Pigp, Letmd1, Slco2b1, Marc1, Epn2, Tprn, Ppp2r5c, Tbc1d14, Gskip, Pdhb, Hif1an, Rbm23, Adipor1, Hectd2, Serpine1, Zdhhc20, Exoc7, Fgf5, Mecp2, Tmem33, Cpne2, Msh2, Dnajb12, Cdkn1a, Lsm11, Plat, Mrpl17, Atg4b, Srsf1, C6orf120, Tspan9, Stx7, Wdr81, Tia1, Gng12, Loxl1, Tmem214, Ddx54, Ipo7, Rbm3, Ablim1, Cyp26b1, Gfpt1, Hn1l, Mtpn, Akap2, Palm2-Akap2, Cxxc5, Taz, Parp1, Inhba, Ptrhd1, Sirpa, Cdh2, Papola, Tmem30a, C1orf50, Eif2b2, Cmc1, Apoa1, Ebf4, Cav2, Txndc5, Picalm, Slc25a4, Ece1, Capza1, C21orf33, Nfib, Commd9, Mppe1, Hddc3, Sorbs3, Prrg1, Ralgapb, Slc25a29, Frmd8, Arf3, Kmt2a, Smim12, Fn1, Manba, Rragc, Tsc22d2, Styxl1, Limch1, Ccng2, Vezt, Etnk1, Slc7a6, Med14, Trub1, Fbxo21, Galnt11, Lmcd1, Necap2, Pdlim5, Gclc |
| DIANA | | Tardbp, Ralgapb, Sema4a, Elavl3, Gclc, 4930455H04Rik, Tmem241, Rc3h1, Hes7, Vegfa, Jrkl, Zmat3, Tfap2e, Tfap2c, Sp100, Nos1, Mier1, Hnrnpu, Txndc17, Eif5, Fam169a, Nbr1, Abcg1, Srsf3, Gm6483, Daam1, Uqcc1, Dnajc3, Tmem67, Sowaha, Slc26a9, Zdhhc3, Foxp2, Trappc13, Ubqln1, 1700017N19Rik, Nfia, Ube2k, Slc25a41, Spam1, Otud4, Olfr112, Rbp4, Mtpn, Spop, Pip5k1a |
| miRDB | | Foxp2, Sema4a, Rc3h1, Crlf3, Sele, Lrp4, Suv420h1, Ubqln1, Ptpn4, Gpr133, Heyl, Nfia, Vapb, Clec4d, Cxxc5, Spop, Csmd1, Ralgapb, Scn3a, Gria4, Faxc, Rab31 |
| (miR -29b-3p) | | |
| TargetScan | | Col1a1, Adamts2, Sparc, Col2a1, Spns1, Ifi30 |
| DIANA | | Tet2, Adamts10, Igf1, Fbn1, Col4a5, Col7a1, Sestd1, Nsd1, Col1a1, Col5a3, C77370, Mst1, Lsm11, Nav1, Col5a2, Hif3a, Trib2, Col5a1, Tet1, Dot1l, Pi15, Dnmt3a, Nfia |
| miRDB | | Tet3, Eln, Atp1b4, Col4a5, Nfia, Brwd3, Robo1, Lysmd1, Tet2, Col11a1, Adamts17, Hdac4, Col5a3, Adamts2, Col4a3, Stard8, Col4a2, Vegfa, Tfeb, Grip1, Zdhhc21, Col9a1, Rnf19a, Hormad1, Has3, Kif26a, Tll1, Klhl28, Col15a1, Pcdhac2, Fbn1, Rab30, Atad2b, Cldn1, Col3a1, Zbtb34, Eomes, Col7a1, Tnfrsf1a, Gm14680, Ifi30, Col5a2, Purg, Pdik1l, Pxdn, Ireb2, Hbp1, Tmem254a, Elf2, Crispld1, Rest, Col5a1, Vash1, Lims1, Eml4, Pcdhac1, Pcdha11, Pcdha12, Trim63, Pcdha9, Pcdha2, Pcdha3, Pcdha1, Pcdha7, Pcdha6, Kdelc1, Pcdha4, Pcdha5, Adamts18, Usp34, Zbtb5, Ypel2, Tet1, Sestd1, Dnmt3a, Nav2, Tpk1, Ccnyl1, Map6, Ppic, Ccser2, Dgkd, Bach2, Rlf, N4bp2l1, Mapre2, Akt3, Bdh1, Col19a1, Snx8, Fermt2, Stag2, Taf5, Lamc1, Slc5a8, Kif26b, Igf1, Nasp, Dicer1, Tdg, C1qtnf6, Sypl2, Tubb2a, Phldb3, B630005N14Rik, Gpr37, Shroom2, Otub2, Cd276, Sgk1, Rnd3, Col4a1, Col6a3, Ybey, Col2a1, Nav1, Mex3b, Slc31a1, Nanp, Ankrd13b, Lrrc16a, Ammecr1l, Traf4, Larp4b, Slc4a7, Hrk, Dgkh, Arrdc3, C77370, Eif4e2, Zfp651, Proser1, Chsy1, Nsd1, Sh3rf3, Dcaf10, 1810013L24Rik, Ppm1d, Tfec, Smek2, Zfp282, Hecw1, Eml6, Adam19, Col22a1, Npas3, Col4a6, Jmy, 9530068E07Rik, Adam12, Cuedc1, Lox, Dio2, Ubn1, Cnr1, Slc25a3, D630045J12Rik, Emp2, Rnpepl1, Fem1b, Psma3, Iffo1, Atrn, Rapgefl1, Chfr, 0610030E20Rik, Pmp22, Birc2, Pten, Tmem183a, Pgap2, Zbtb10, Ap4e1, Ddx3x, Cdca4, Mycn, Nckap5, Trafd1, Jarid2, Morf4l2, Wisp1, C030046E11Rik, Snx24, Dcx, Xkr7, Dpysl5, Elovl4, Fam168b, Enpp6, Gtpbp2, Eml5, Clock, Ap1g1, Ythdf3, Mlf1, Zfp36l1, Stx16, Dusp2, Gxylt2, Blmh, Slc16a1, Sh3pxd2a, Rarb, Adamts9, Col26a1, Rnf138, Vcl, Zdhhc5, Klhdc3, Lif, Abce1, Slc43a2, Pan2, Pcdha10, Dcun1d4, Arpp19, Cmpk1, Rmnd5a, Gm17296, Pcdha8, Dennd1b, Hmgcr, Kctd15, Pcgf3, Elmo2, Adamts10, Atp5g1, Rfx7, Erlin2, Appl1, Lamtor1, Ankrd49, Taf7, Vash2, Wbp1l, Ptbp3, Smpd3, Abhd5, Ddx46, Per3 |
